# Supplementary material for: Relationship between Solitary Masturbation and Sexual Satisfaction: A Systematic Review
Source: Healthcare (Basel). 2024 Jan 17;12(2):235. doi: 10.3390/healthcare12020235 (PMC10815145; doi:10.3390/healthcare12020235)
Supplement: Supplementary file 1 [file healthcare-12-00235-s001.zip › healthcare-2807405-supplementary.pdf]

Supplementary Data S1.

N.	Title	Authors	Year
1	Masturbation Among Malaysian Young Adults: Associated Sexual and Psychological Well-Being Outcomes	Phuah L.A.; Teng J.H.J.; Goh P.H.	2023
2	Online Pornography Use and Sexual Satisfaction in Association With Relationship Satisfaction Among Middle-Aged and Older People	Sevciková, A; Gocieková, V; Stasek, A; Gottfried, J; Daneback, K	2023
3	Masturbation Among Malaysian Young Adults: Associated Sexual and Psychological Well-Being Outcomes	Phuah, L; Teng, JHJ; Goh, PH	2023
4	The Role of Mutual Masturbation within Relationships: Associations with Sexual Satisfaction and Sexual Self-Esteem	Kiliç, D; Armstrong, HL; Graham, CA	2023
5	Singles Not Sexually Satisfied? Prevalence and Predictors of Sexual Satisfaction in Single versus Partnered Adults	Fischer, N	2023
6	Sexual Risk Behavior and Satisfaction Among Men Who Have Sex With Men Living With Detectable HIV Viral Loads: A Nationwide Online Survey in China	Fu, LW; Wang, BY; Tian, T; Zhou, XY; Lu, Z; Sun, YH; Zheng, WR; Gao, YX; Lin, YF; Li, H; Zou, HC	2023
7	The sex life of male patients with cirrhosis and its organic factors: What we have got so far?	Darmadi, D; Pakpahan, C; Ruslie, RH; Amanda, B; Ibrahim, R	2023
8	Sexual Function, Behavior, and Satisfaction in Masters Athletes	Wang, TY; Heath, MA; Tanaka, SK; Tanaka, H	2023
9	The Longer it is, the Closer One Feels: Perception of Emotional Closeness to the Partner, Relationship Duration, Sexual Activity, and Satisfaction in Married and Cohabiting Persons in Norway	Træen, B; Kvalem, IL	2023
10	Not Who You Are, But Who You Are With: Re-examining Women's Less Satisfying Sexual Debuts	Peragine, DE; Kim, JJ; Maxwell, JA; Skorska, MN; Impett, EA; Cunningham, WA; Vanderlaan, DP	2023
11	Factors influencing satisfaction with male circumcision in Taiwan	Chen, CH; Cheng, WM; Fan, YH; Chang, TP	2023
12	Changes in sexuality and couple relationship during the COVID-19 lockdown in Portugal: findings from an online survey	Vieira, AS; Santos, HC; Pereira, J; Madeira, L	2023

13	Patterns of Genital and Subjective Sexual Arousal in Cisgender Asexual Men	Skorska, MN; Yule, MA; Bogaert, AF; Brotto, LA	2023
14	Characteristics of men who report symptoms of delayed ejaculation: providing support for empirically derived diagnostic criteria	Rowland, DL; Attinger, DR; Morrow, AL; Motofei, I; Hevesi, K	2023
15	The Role of Mutual Masturbation within Relationships: Associations with Sexual Satisfaction and Sexual Self-Esteem	Kılıç, Dilan;Armstrong, Heather L;Graham, Cynthia A	2023
16	The role of mutual masturbation within relationships: Associations with sexual satisfaction and sexual self-esteem	Kılıç, Dilan;Armstrong, Heather L.;Graham, Cynthia A.	2023
17	Not who you are, but who you are with: Re-examining women's less satisfying sexual debuts	Peragine, Diana E.;Kim, James J.;Maxwell, Jessica A.;Skorska, Malvina N.;Impett, Emily A.;Cunningham, William A.;VanderLaan, Doug P.	2023
18	Changes in sexuality and couple relationship during the covid-19 lockdown in portugal: Findings from an online survey	Vieira, Ana Sofia;Castro Santos, Henrique;Pereira, Joana;Madeira, Luís	2023
19	The Longer it is, the Closer One Feels: Perception of Emotional Closeness to the Partner, Relationship Duration, Sexual Activity, and Satisfaction in Married and Cohabiting Persons in Norway	Træen, Bente;Kvalem, Ingela Lundin	2023
20	Masturbation Among Malaysian Young Adults: Associated Sexual and Psychological Well-Being Outcomes.	Phuah, Li Ann;Teng, Jaclyn Hui Jie;Goh, Pei Hwa	2023
21	Characteristics of men who report symptoms of delayed ejaculation: Providing support for empirically derived diagnostic criteria	Rowland, David L.;Attinger, Drew R.;Morrow, Abigail L.;Motofei, Ion;Hevesi, Krisztina	2023
22	Changes in sexual activities, function, and satisfaction during the COVID-19 pandemic era: a systematic review and meta-analysis.	Qaderi, Kowsar;Yazdkhasti, Mansoureh;Zangeneh, Sanaz;Behbahani, Bahar Morshed;Kalhor, Mehri;Shamsabadi, Ahmadreza;Jesmani, Younes;Norouzi, Solmaz;Kajbafvala, Mehrnaz;Khodavirdilou, Rasa;Rahmani, Nahid;Namadian, Masoumeh;Ezabadi, Sajjad Ghane;Alkatout, Ibrahim;Mehraeen, Esmaeel;Rasoal, Dara	2023

23	Characteristics of men who report symptoms of delayed ejaculation: providing support for empirically derived diagnostic criteria.	Rowland, David L;Attinger, Drew R;Morrow, Abigail L;Motofei, Ion;Hevesi, Krisztina	2023
24	The sex life of male patients with cirrhosis and its organic factors: What we have got so far?	Darmadi, Darmadi;Pakpahan, Cennikon;Riska Habriel Ruslie;Bella, Amanda;Ibrahim, Raditya	2023
25	Patterns of Genital and Subjective Sexual Arousal in Cisgender Asexual Men	Skorska, Malvina N;Yule, Morag A;Bogaert, Anthony F;Brotto, Lori A	2023
26	Patterns of Genital and Subjective Sexual Arousal in Cisgender Asexual Men.	Skorska, Malvina N;Yule, Morag A;Bogaert, Anthony F;Brotto, Lori A	2023
27	Sexual Function, Behavior, and Satisfaction in Masters Athletes	Wang, Tianyu;Heath, Melanie A;Tanaka, Sebastian K;Tanaka, Hirofumi	2023
28	The sex life of male patients with cirrhosis and its organic factors: What we have got so far?	Darmadi, Darmadi;Pakpahan, Cennikon;Ruslie, Riska Habriel;Amanda, Bella;Ibrahim, Raditya	2023
29	Do pornography use and masturbation play a role in erectile dysfunction and relationship satisfaction in men?	Rowland D.L.; Castleman J.M.; Bacys K.R.; Csonka B.; Hevesi K.	2023
30	Sexual Risk Behavior and Satisfaction Among Men Who Have Sex With Men Living With Detectable HIV Viral Loads: A Nationwide Online Survey in China	Fu L.; Wang B.; Tian T.; Zhou X.; Lu Z.; Sun Y.; Zheng W.; Gao Y.; Lin Y.-F.; Li H.; Zou H.; Van Der Veen S.	2023
31	Let's Talk about Sex… and ADHD: Findings from an Anonymous Online Survey	Young S.; Klassen L.J.; Reitmeier S.D.; Matheson J.D.; Gudjonsson G.H.	2023
32	The sex life of male patients with cirrhosis and its organic factors: What we have got so far?	Darmadi D.; Pakpahan C.; Ruslie R.H.; Amanda B.; Ibrahim R.	2023
33	The influence of cannabis on sexual functioning and satisfaction	Moser A.; Ballard S.M.; Jensen J.; Averett P.	2023
34	A case-control study on depression, anxiety, and belief in sexual myths in trans women	Uyar B.; Yucel I.; Uyar E.; Ateş Budak E.; Kelle I.; Bulbuloglu S.	2023
35	The Role of Mutual Masturbation within Relationships: Associations with Sexual Satisfaction and Sexual Self-Esteem	Kılıç D.; Armstrong H.L.; Graham C.A.	2023

36	Sexual activity and function of adult men with spina bifida	Szymanski K.M.; Roth J.D.; Hensel D.J.; Wiener J.S.; Younsi N.; Stein R.; Whittam B.; Kaefer M.; Rink R.C.; Cain M.P.; Misseri R.	2023
37	Not Who You Are, But Who You Are With: Re-examining Women's Less Satisfying Sexual Debuts	Peragine D.E.; Kim J.J.; Maxwell J.A.; Skorska M.N.; Impett E.A.; Cunningham W.A.; VanderLaan D.P.	2023
38	Mediators of Change in Cognitive-Behavioral Couple Therapy for Genito-Pelvic Pain: Results of a Randomized Clinical Trial	Santerre-Baillargeon M.; Rosen N.O.; Vaillancourt-Morel M.-P.; Corsini-Munt S.; Steben M.; Mayrand M.-H.; Bergeron S.	2023
39	Sexual Function, Behavior, and Satisfaction in Masters Athletes	Wang T.; Heath M.A.; Tanaka S.K.; Tanaka H.	2023
40	Characteristics of men who report symptoms of delayed ejaculation: providing support for empirically derived diagnostic criteria	Rowland D.L.; Attinger D.R.; Morrow A.L.; Motofei I.; Hevesi K.	2023
41	Changes in sexuality and couple relationship during the COVID-19 lockdown in Portugal: findings from an online survey	Vieira A.S.; Castro Santos H.; Pereira J.; Madeira L.	2023
42	Sexuality and fertility desire in a large cohort of individuals with 46, XY differences in sex development	Batista R.L.; Inácio M.; Brito V.N.; Sircili M.H.P.; Bag M.J.; Gomes N.L.; Costa E.M.F.; Domenice S.; Mendonca B.B.	2023
43	Patterns of Genital and Subjective Sexual Arousal in Cisgender Asexual Men	Skorska M.N.; Yule M.A.; Bogaert A.F.; Brotto L.A.	2023
44	Sexual satisfaction and sexual behaviors during the COVID-19 pandemic: results from the International Sexual Health And REproductive (I-SHARE) health survey in Luxembourg	Fischer, VJ; Bravo, RG; Brunnet, AE; Michielsen, K; Tucker, JD; Campbell, L; Vögele, C	2022
45	A Seemingly Paradoxical Relationship Between Masturbation Frequency and Sexual Satisfaction	Fischer, N; Træen, B	2022
46	Prevalence of Masturbation and Associated Factors Among Older Adults in Four European Countries	Fischer, N; Graham, CA; Træen, B; Hald, GM	2022
47	BDSM: Does it Hurt or Help Sexual Satisfaction, Relationship Satisfaction, and Relationship Closeness?	Strizzi, JM; Overup, CS; Cipric, A; Hald, GM; Træen, B	2022
48	Relationships of Frequency, Secrecy, and Attitude Toward Pornography Use with the Sexual Satisfaction of Couples	del Re, UB; Hilpert, P; Bodenmann, G	2022

49	The Influence of Types of Stimulation and Attitudes to Clitoral Self-stimulation on Female Sexual and Orgasm Satisfaction: a Cross-sectional Study	Hoy, M; van Stein, K; Strauss, B; Brenk-Franz, K	2022
50	First Adolescent Romantic and Sexual Experiences in Individuals With Differences of Sex Development/Intersex Conditions	de Brouwer, IJ; Suijkerbuijk, M; van de Grift, TC; Kreukels, BPC	2022
51	Changes in the Sexual Behavior of Partners in Each Trimester of Pregnancy in Otwock in Polish Couples	Kulhawik, R; Zborowska, K; Grabarek, BO; Boron, D; Skrzypulec-Plinta, V; Drosdzol-Cop, A	2022
52	Sexual and Reproductive Health during the COVID-19 Pandemic: Results from a Cross-Sectional Online Survey in Germany	Raeuchle, J; Briken, P; Schroeder, J; Ivanova, O	2022
53	Premature Ejaculation Measures During Partnered Sex and Masturbation: What These Findings Tell Us About the Nature and Rigidity of Premature Ejaculation	Rowland, DL; Teague, LG; Hevesi, K	2022
54	Do Pornography Use and Masturbation Frequency Play a Role in Delayed/Inhibited Ejaculation during Partnered Sex? A Comprehensive and Detailed Analysis	Rowland, DL; Morrow, AL; Hamilton, BD; Hevesi, K	2022
55	Satisfaction with Singlehood and Sexual Activity	Træen, B; Kvalem, IL	2022
56	Masturbation Frequency and Sexual Function in Individuals with and without Sexual Partners	Huang, SJ; Niu, CY; Santtila, P	2022
57	Virtual Reality Versus Flatscreen Pornography: Correlations and Effects on Relationship Satisfaction, Rape-Myth Acceptance, and Problematic Use	Mitchell, KM; Ratan, R; Maas, MK; Holt, K; Slaker, JS; Gambino, A	2022
58	Masturbatory Behavior and Body Image: A Study Among Brazilian Women	de Lima, TEO; Dissenha, RP; Skare, TL; Leinig, CAS	2022
59	Focusing the Conceptualization of Erotophilia and Erotophobia on Global Attitudes Toward Sex: Development and Validation of the Sex Positivity-Negativity Scale	Hangen, F; Rogge, RD	2022
60	Impacts of COVID-19 on sexual behaviour in Britain: findings from a large, quasi-representative survey (Natsal-COVID)	Mercer, CH; Clifton, S; Riddell, J; Tanton, C; Freeman, L; Copas, AJ; Dema, E; Pérez, RB; Gibbs, J; Macdowall, W;	2022

		Menezes, D; Ridge, MC; Bonell, C; Sonnenberg, P; Field, N; Mitchell, KR	
61	First adolescent romantic and sexual experiences in individuals with differences of sex development/intersex conditions	de Brouwer, Iris J.;Suijkerbuijk, Merel;van de Grift, Tim C.;Kreukels, Baudewijntje P. C.	2022
62	First Adolescent Romantic and Sexual Experiences in Individuals With Differences of Sex Development/Intersex Conditions.	de Brouwer, Iris J;Suijkerbuijk, Merel;van de Grift, Tim C;Kreukels, Baudewijntje P C	2022
63	Sexual function, behavior, and satisfaction in masters athletes	Wang, Tianyu;Heath, Melanie A.;Tanaka, Sebastian K.;Tanaka, Hirofumi	2022
64	Impacts of COVID-19 on sexual behaviour in Britain: findings from a large, quasi-representative survey (Natsal-COVID)	Mercer, Catherine H;Soazig Clifton;Riddell, Julie;Tanton, Clare;Freeman, Lily;Copas, Andrew J;Dema, Emily;Pérez, Raquel Bosó;Gibbs, Jo;Macdowall, Wendy;Menezes, Dee;Ridge, Mary-Clare;Bonell, Chris;Sonnenberg, Pam;Field, Nigel;Mitchell, Kirstin R	2022
65	Impacts of COVID-19 on sexual behaviour in Britain: findings from a large, quasi-representative survey (Natsal-COVID).	Mercer, Catherine H;Clifton, Soazig;Riddell, Julie;Tanton, Clare;Freeman, Lily;Copas, Andrew J;Dema, Emily;Bosó Pérez, Raquel;Gibbs, Jo;Macdowall, Wendy;Menezes, Dee;Ridge, Mary-Clare;Bonell, Chris;Sonnenberg, Pam;Field, Nigel;Mitchell, Kirstin R	2022
66	Satisfaction with singlehood and sexual activity	Træen, Bente;Kvalem, Ingela Lundin	2022
67	2022-RA-570-ESGO Gynecological cancer treatment and couple's sexuality	Nowosielski, Krzysztof;Wróbel, Beata;Pałka, Aleksandra;Dąbrowski, Filip	2022
68	Satisfaction with Singlehood and Sexual Activity	Træen, Bente;Kvalem, Ingela Lundin	2022
69	The Influence of Types of Stimulation and Attitudes to Clitoral Self-stimulation on Female Sexual and Orgasm Satisfaction: a Cross-sectional Study	Hoy, Madita;van Stein, Katharina;Strauss, Bernhard;Brenk-Franz, Katja	2022
70	A Seemingly Paradoxical Relationship Between Masturbation Frequency and Sexual Satisfaction	Fischer, Nantje;Træen, Bente	2022

71	A Seemingly Paradoxical Relationship Between Masturbation Frequency and Sexual Satisfaction.	Fischer, Nantje;Træen, Bente	2022
72	A seemingly paradoxical relationship between masturbation frequency and sexual satisfaction	Fischer, Nantje;Træen, Bente	2022
73	Sexual satisfaction and sexual behaviors during the COVID-19 pandemic: results from the International Sexual Health And REproductive (I-SHARE) health survey in Luxembourg.	Fischer, Vinicius Jobim;Bravo, Raquel Gómez;Brunnet, Alice Einloft;Michielsen, Kristien;Tucker, Joseph D;Campbell, Linda;Vögele, Claus	2022
74	The impact of the COVID-19 pandemic on sexual health in CIS women living in Germany	Batz, Falk;Lermer, Eva;Hatzler, Laura;Vilsmaier, Theresa;Schröder, Lennard;Chelariu-Raicu, Anca;Behr, Joachim;Mahner, Sven;Buspavanich, Pichit;Thaler, Christian J.	2022
75	The Impact of the COVID-19 Pandemic on Sexual Health in Cis Women Living in Germany.	Batz, Falk;Lermer, Eva;Hatzler, Laura;Vilsmaier, Theresa;Schröder, Lennard;Chelariu-Raicu, Anca;Behr, Joachim;Mahner, Sven;Buspavanich, Pichit;Thaler, Christian J	2022
76	Associations between tactile intimacy and sleep quality in healthy adults: A systematic review	Dueren, Anna L.;Perach, Rotem;Banissy, Jasmine F. M.;Bowling, Natalie C.;Gregory, Alice M.;Banissy, Michael J.	2022
77	Associations between tactile intimacy and sleep quality in healthy adults: A systematic review.	Dueren, Anna L;Perach, Rotem;Banissy, Jasmine F M;Bowling, Natalie C;Gregory, Alice M;Banissy, Michael J	2022
78	Patterns of genital and subjective sexual arousal in cisgender asexual men	Skorska, Malvina N.;Yule, Morag A.;Bogaert, Anthony F.;Brotto, Lori A.	2022
79	Masturbatory behavior and body image: A study among Brazilian women	de Lima, Taís Elena Oliveira;Dissenha, Rafaela Piccinelli;Skare, Thelma Larocca;Leinig, Cezar Augusto Soares	2022
80	Prevalence of Masturbation and Associated Factors Among Older Adults in Four European Countries	Fischer Nantje;Graham, Cynthia A;Træen Bente;Hald, Gert Martin	2022
81	Masturbatory Behavior and Body Image: A Study Among Brazilian Women	de Lima Taís Elena Oliveira;Piccinelli, Dissenha Rafaela;Skare, Thelma Larocca;Leinig Cezar Augusto Soares	2022

82	Prevalence of Masturbation and Associated Factors Among Older Adults in Four European Countries.	Fischer, Nantje;Graham, Cynthia A;Træen, Bente;Hald, Gert Martin	2022
83	Changes in the Sexual Behavior of Partners in Each Trimester of Pregnancy in Otwock in Polish Couples.	Kulhawik, Robert;Zborowska, Katarzyna;Grabarek, Beniamin Oskar;Boroń, Dariusz;Skrzypulec-Plinta, Violetta;Drosdzol-Cop, Agnieszka	2022
84	Sexual and Reproductive Health during the COVID-19 Pandemic: Results from a Cross-Sectional Online Survey in Germany.	Räuchle, Jule;Briken, Peer;Schröder, Johanna;Ivanova, Olena	2022
85	<i>Reinventing Licentiousness: Pornography and Modern China</i> by Y. Yvon Wang (review)	Rocha, Leon Antonio	2022
86	Premature Ejaculation Measures During Partnered Sex and Masturbation: What These Findings Tell Us About the Nature and Rigidity of Premature Ejaculation	Rowland, David L;Teague, Lijana G;Hevesi, Krisztina	2022
87	Exploring the adult sexual wellbeing and behavior during the COVID-19 pandemic. A systematic review and meta-analysis.	Mourikis, Iraklis;Kokka, Ioulia;Koumantarou-Malisiova, Elli;Kontoangelos, Konstantinos;Konstantakopoulos, George;Papageorgiou, Charalabos	2022
88	Masturbation Frequency and Sexual Function in Individuals with and without Sexual Partners	Huang, Sijia;Niu, Caoyuan;Santtila, Pekka	2022
89	Sexual satisfaction and sexual behaviors during the COVID-19 pandemic: results from the International Sexual Health And REproductive (I-SHARE) health survey in Luxembourg	Fischer, Vinicius Jobim;Raquel Gómez Bravo;Brunnet, Alice Einloft;Michielsen, Kristien;Tucker, Joseph D;Campbell, Linda;Vögele, Claus	2022
90	Do Pornography Use and Masturbation Frequency Play a Role in Delayed/Inhibited Ejaculation during Partnered Sex? A Comprehensive and Detailed Analysis	Rowland, David L;Morrow, Abigail L;Hamilton, Benjamin D;Hevesi, Krisztina	2022
91	Is Basson's model of sexual response relevant? A commentary	Balon, Richard	2022
92	Changes in the Sexual Behavior of Partners in Each Trimester of Pregnancy in Otwock in Polish Couples	Kulhawik, Robert;Zborowska, Katarzyna;Grabarek, Beniamin Oskar;Boroń, Dariusz;Skrzypulec-Plinta, Violetta;Drosdzol-Cop, Agnieszka	2022

93	Premature ejaculation measures during partnered sex and masturbation: What these findings tell us about the nature and rigidity of premature ejaculation	Rowland, David L.;Teague, Lijana G.;Hevesi, Krisztina	2022
94	Premature Ejaculation Measures During Partnered Sex and Masturbation: What These Findings Tell Us About the Nature and Rigidity of Premature Ejaculation.	Rowland, David L;Teague, Lijana G;Hevesi, Krisztina	2022
95	Focusing the Conceptualization of Erotophilia and Erotophobia on Global Attitudes Toward Sex: Development and Validation of the Sex Positivity–Negativity Scale	Hangen Forrest;Rogge, Ronald D	2022
96	Sexual and Reproductive Health during the COVID-19 Pandemic: Results from a Cross-Sectional Online Survey in Germany	Räuchle, Jule;Briken, Peer;Schröder, Johanna;Ivanova, Olena	2022
97	Focusing the Conceptualization of Erotophilia and Erotophobia on Global Attitudes Toward Sex: Development and Validation of the Sex Positivity-Negativity Scale.	Hangen, Forrest;Rogge, Ronald D	2022
98	Single and partnered individuals' sexual satisfaction as a function of sexual desire and activities: Results using a sexual satisfaction scale demonstrating measurement invariance across partnership status.	Park, Y.; MacDonald, G.	2022
99	Sexual satisfaction and sexual behaviors during the COVID-19 pandemic: results from the International Sexual Health And REproductive (I-SHARE) health survey in Luxembourg	Fischer V.J.; Bravo R.G.; Brunnet A.E.; Michielsen K.; Tucker J.D.; Campbell L.; Vögele C.	2022
100	Changes in the Sexual Behavior of Partners in Each Trimester of Pregnancy in Otwock in Polish Couples	Kulhawik R.; Zborowska K.; Grabarek B.O.; Boroń D.; Skrzypulec-Plinta V.; Drosdzol-Cop A.	2022
101	First Adolescent Romantic and Sexual Experiences in Individuals With Differences of Sex Development/Intersex Conditions	de Brouwer I.J.; Suijkerbuijk M.; van de Grift T.C.; Kreukels B.P.C.	2022
102	Use of dental drill handpiece to remove steel nut causing penile strangulation: a case report and review of the literature	Nguyen T.T.; Ngo X.T.; Chau Q.T.; Hoang K.C.; Dinh L.Q.V.; Ly H.T.; Hoang T.D.; Dobbs R.W.; Thai M.S.	2022

103	Focusing the Conceptualization of Erotophilia and Erotophobia on Global Attitudes Toward Sex: Development and Validation of the Sex Positivity–Negativity Scale	Hangen F.; Rogge R.D.	2022
104	Prevalence of Masturbation and Associated Factors Among Older Adults in Four European Countries	Fischer N.; Graham C.A.; Træen B.; Hald G.M.	2022
105	Are the Criteria for the Diagnosis of Premature Ejaculation Applicable to Gay Men or Sexual Activities Other than Penile-Vaginal Intercourse?	McNabney S.M.; Weseman C.E.; Hevesi K.; Rowland D.L.	2022
106	The Impact of the COVID-19 Pandemic on Sexual Health in Cis Women Living in Germany	Batz F.; Lermer E.; Hatzler L.; Vilsmaier T.; Schröder L.; Chelariu-Raicu A.; Behr J.; Mahner S.; Buspavanich P.; Thaler C.J.	2022
107	Sexual and Reproductive Health during the COVID-19 Pandemic: Results from a Cross-Sectional Online Survey in Germany	Räuchle J.; Briken P.; Schröder J.; Ivanova O.	2022
108	Satisfaction with Singlehood and Sexual Activity	Træen B.; Kvalem I.L.	2022
109	Masturbatory Behavior and Body Image: A Study Among Brazilian Women	de Lima T.E.O.; Dissenha R.P.; Skare T.L.; Leinig C.A.S.	2022
110	RUNX1 is a promising prognostic biomarker and related to immune infiltrates of cancer-associated fibroblasts in human cancers	Tuo Z.; Zhang Y.; Wang X.; Dai S.; Liu K.; Xia D.; Wang J.; Bi L.	2022
111	Premature Ejaculation Measures During Partnered Sex and Masturbation: What These Findings Tell Us About the Nature and Rigidity of Premature Ejaculation	Rowland D.L.; Teague L.G.; Hevesi K.	2022
112	Impacts of COVID-19 on sexual behaviour in Britain: findings from a large, quasi-representative survey (Natsal-COVID)	Mercer C.H.; Clifton S.; Riddell J.; Tanton C.; Freeman L.; Copas A.J.; Dema E.; Pérez R.B.; Gibbs J.; Macdowall W.; Menezes D.; Ridge M.-C.; Bonell C.; Sonnenberg P.; Field N.; Mitchell K.R.	2022
113	Orgasm Rating Scale and Bodily Sensations of Orgasm Scale: Validation for Use With Pre, Peri, and Post-Menopausal Women	Webb A.E.; Reissing E.D.; Huta V.	2022

114	Changes in sexual functions and habits of healthcare workers during the ongoing COVID-19 outbreak: a cross-sectional survey study	Güzel A.; Döndü A.	2022
115	Functional and Oncological Outcomes of Female Pelvic Organ–preserving Robot-assisted Radical Cystectomy	Lavallée E.; Dovey Z.; Pathak P.; Dey L.; Renström Koskela L.; Hosseini A.; Waingankar N.; Mehrazin R.; Sfakianos J.; Hosseini A.; Wiklund P.	2022
116	Sexual dysfunction associated with chronic retention of foreign bodies in the low urinary tract	Zhang Z.; Zhu X.; Wang Y.; Chen D.; Fan J.; Deng C.; Liu G.; Yang L.; Feloney M.; Wang X.; Zhang Y.	2022
117	The Longer it is, the Closer One Feels: Perception of Emotional Closeness to the Partner, Relationship Duration, Sexual Activity, and Satisfaction in Married and Cohabiting Persons in Norway	Træen B.; Kvalem I.L.	2022
118	A Seemingly Paradoxical Relationship Between Masturbation Frequency and Sexual Satisfaction	Fischer N.; Træen B.	2022
119	Male Reproductive Health-study of a sperm donor population	Fonseca A.C.S.; Barreiro M.; Tomé A.; Vale-Fernandes E.	2022
120	Sexual health outcomes after penile reconstruction in the exstrophy–epispadias complex	Harris T.G.W.; Khandge P.; Wu W.J.; Leto Barone A.A.; Manyevitch R.; Sholklapper T.; Bivalacqua T.J.; Burnett A.L.; Redett R.J., III; Gearhart J.P.	2022
121	The Big O: Sociocultural Influences on Orgasm Frequency and Sexual Satisfaction in Women	Lentz, AM; Zaikman, Y	2021
122	Uncovering the Most Important Factors for Predicting Sexual Desire Using Explainable Machine Learning	Vowels, LM; Vowels, MJ; Mark, KP	2021
123	Impact of COVID-19 Pandemic on the Sexual Function of Health Professionals From an Epicenter in Brazil	Neto, RP; Nascimento, BCG; Silva, GCD; Barbosa, JABA; de Bessa, J; Teixeira, TA; Srougi, M; Nahas, WC; Hallak, J; Cury, J	2021
124	Pornography Use and Sexual Health among Same-Sex and Mixed-Sex Couples: An Event-Level Dyadic Analysis	Vaillancourt-Morel, MP; Rosen, NO; Stulhofer, A; Bosisio, M; Bergeron, S	2021
125	Hormonal Contraception and Sexuality: Causal Effects, Unobserved Selection, or Reverse Causality?	Botzet, LJ; Gerlach, TM; Driebe, JC; Penke, L; Arslan, RC	2021

126	Partnership status, living arrangements, and changes in sexual behaviour and satisfaction during the COVID-19 lockdown: insights from an observational, cross-sectional online survey in Singapore	Tan, RKJ; O'Hara, CA; Kumar, N	2021
127	Pornography and Sexual Dissatisfaction: The Role of Pornographic Arousal, Upward Pornographic Comparisons, and Preference for Pornographic Masturbation	Wright, PJ; Paul, B; Herbenick, D; Tokunaga, RS	2021
128	Misconceptions About Sexual Health in Older Women: Why We Need to Talk About It	Kuhle, CL; Zhang, X; Kapoor, E	2021
129	Developing a prediction model for the self-evaluation of erectile dysfunction in an adult male population	Cai, ZL; Li, HJ	2021
130	Sexual and psychological health of couples with azoospermia in the context of the COVID-19 pandemic	Dong, M; Tao, YQ; Wu, SS; Li, ZT; Wang, XB; Tan, JC	2021
131	Sexual Quality of Life in Men <60 Years Old after Coronary Bypass Surgery	Ghazy, T; Haeberle, E; Kappert, U; Petzold, S; Plötze, K; Mashhour, A; Matschke, K; Ouda, A	2021
132	Faking Orgasm: Relationship to Orgasmic Problems and Relationship Type in Heterosexual Women	Hevesi, K; Horvath, Z; Sal, D; Miklos, E; Rowland, DL	2021
133	Penile ring entrapment and strangulation: A case report at Kampala International University Teaching Hospital in Western Uganda	Kyomukama, LA; Ssebuufu, R; Wani, SA; Waziri, MA; Lule, H	2021
134	The Impact of the COVID-19 Pandemic on Sexual Behaviors: Findings From a National Survey in the United States	Gleason, N; Banik, S; Braverman, J; Coleman, E	2021
135	How the COVID-19 Pandemic Affects Sexual Behavior of Hetero-, Homo-, and Bisexual Males in Germany	Mumm, JN; Vilsmaier, T; Schuetz, JM; Rodler, S; Zehni, AZ; Bauer, RM; Staehler, M; Stief, CG; Batz, F	2021
136	Single and multiple orgasm experience among women in heterosexual partnerships. Results of the German Health and Sexuality Survey (GeSiD)	Cerwenka, Susanne;Dekker, Arne;Pietras, Laura;Briken, Peer	2021

137	Single and Multiple Orgasm Experience Among Women in Heterosexual Partnerships. Results of the German Health and Sexuality Survey (GeSiD).	Cerwenka, Susanne;Dekker, Arne;Pietras, Laura;Briken, Peer	2021
138	Prevalence of masturbation and associated factors among older adults in four european countries	Fischer, Nantje;Graham, Cynthia A.;Træen, Bente;Hald, Gert Martin	2021
139	The impact of the COVID-19 pandemic on sexual behaviors: Findings from a national survey in the United States	Gleason, Neil;Banik, Swagata;Braverman, Jesse;Coleman, Eli	2021
140	Partnership status, living arrangements, and changes in sexual behaviour and satisfaction during the COVID-19 lockdown: insights from an observational, cross-sectional online survey in Singapore.	Tan, Rayner Kay Jin;O'Hara, Caitlin Alsandria;Kumar, Navin	2021
141	The Impact of the COVID-19 Pandemic on Sexual Behaviors: Findings From a National Survey in the United States.	Gleason, Neil;Banik, Swagata;Braverman, Jesse;Coleman, Eli	2021
142	Hormonal contraception and sexuality: Causal effects, unobserved selection, or reverse causality?	Botzet, Laura J.;Gerlach, Tanja M.;Driebe, Julie C.;Penke, Lars;Arslan, Ruben C.	2021
143	Sexual and psychological health of couples with azoospermia in the context of the COVID-19 pandemic	Meng Dong;Tao, Yanqiang;Wu, Shanshan;Li, Zhengtao;Wang, Xiaobin;Tan, Jichun	2021
144	Hormonal Contraception and Sexuality: Causal Effects, Unobserved Selection, or Reverse Causality?	Botzet, Laura J;Gerlach, Tanja M;Driebe, Julie C;Penke Lars;Arslan, Ruben C	2021
145	Paris' medical residents' sexual health before the SARS-CoV-2 burden	Pierre-Emmanuel Cailleaux;Koenig, Manon	2021
146	Paris' medical residents' sexual health before the SARS-CoV-2 burden	Pierre-Emmanuel Cailleaux;Koenig, Manon	2021
147	Impact of COVID-19 Pandemic on the Sexual Function of Health Professionals From an Epicenter in Brazil.	Neto, Rubens Pedrenho;Nascimento, Bruno C G;Carvalho Dos Anjos Silva, Gabriel;Barbosa, João Arthur Brunhara Alves;Júnior, José de Bessa;Teixeira, Thiago Afonso;Srougi, Miguel;Nahas, William Carlos;Hallak, Jorge;Cury, José	2021

148	Focusing the conceptualization of erotophilia and erotophobia on global attitudes toward sex: Development and validation of the sex positivity–negativity scale	Hangen, Forrest;Rogge, Ronald D.	2021
149	How the COVID-19 Pandemic Affects Sexual Behavior of Hetero-, Homo-, and Bisexual Males in Germany.	Mumm, Jan-Niclas;Vilsmaier, Theresa;Schuetz, Julius M;Rodler, Severin;Zati Zehni, Alaleh;Bauer, Ricarda M;Staehler, Michael;Stief, Christian G;Batz, Falk	2021
150	The influence of types of stimulation and attitudes to clitoral self-stimulation on female sexual and orgasm satisfaction: A cross-sectional study	Hoy, Madita;van Stein, Katharina;Strauss, Bernhard;Brenk-Franz, Katja	2021
151	Uncovering the most important factors for predicting sexual desire using explainable machine learning	Vowels, Laura M.;Vowels, Matthew J.;Mark, Kristen P.	2021
152	Uncovering the Most Important Factors for Predicting Sexual Desire Using Explainable Machine Learning.	Vowels, Laura M;Vowels, Matthew J;Mark, Kristen P	2021
153	The big "O": Sociocultural influences on orgasm frequency and sexual satisfaction in women	Lentz, Anna Maree;Zaikman, Yuliana	2021
154	The Big "O": Sociocultural Influences on Orgasm Frequency and Sexual Satisfaction in Women	Lentz, Anna Maree;Zaikman Yuliana	2021
155	Sexual Quality of Life in Men <60 Years Old after Coronary Bypass Surgery.	Ghazy, Tamer;Haeberle, Erwin;Kappert, Utz;Petzold, Stephan;Plötze, Katrin;Mashhour, Ahmed;Matschke, Klaus;Ouda, Ahmed	2021
156	Pornography and sexual dissatisfaction: The role of pornographic arousal, upward pornographic comparisons, and preference for pornographic masturbation	Wright, Paul J.;Paul, Bryant;Herbenick, Debby;Tokunaga, Robert S.	2021
157	Changes in pornography use and sexual behaviour during lockdown	Koós, M;Bőthe, B;Király, O;Paksi, B;Demetrovics, Z	2021
158	Pornography Use and Sexual Health among Same-Sex and Mixed-Sex Couples: An Event-Level Dyadic Analysis	Vaillancourt-Morel Marie-Pier;Rosen, Natalie O;Štulhofer Aleksandar;Bosisio Myriam;Bergeron, Sophie	2021

159	Developing a prediction model for the self-evaluation of erectile dysfunction in an adult male population	Cai, Zhonglin;Li, Hongjun	2021
160	Pornography use and sexual health among same-sex and mixed-sex couples: An event-level dyadic analysis	Vaillancourt-Morel, Marie-Pier;Rosen, Natalie O.;Štulhofer, Aleksandar;Bosisio, Myriam;Bergeron, Sophie	2021
161	Pornography Use and Sexual Health among Same-Sex and Mixed-Sex Couples: An Event-Level Dyadic Analysis.	Vaillancourt-Morel, Marie-Pier;Rosen, Natalie O;Štulhofer, Aleksandar;Bosisio, Myriam;Bergeron, Sophie	2021
162	Developing a prediction model for the self-evaluation of erectile dysfunction in an adult male population.	Cai, Zhonglin;Li, Hongjun	2021
163	Partnership status, living arrangements, and changes in sexual behaviour and satisfaction during the COVID-19 lockdown: insights from an observational, cross-sectional online survey in Singapore	Jin Tan, Rayner Kay;Caitlin Alsandria O'Hara;Kumar, Navin	2021
164	Sexual and psychological health of couples with azoospermia in the context of the COVID-19 pandemic.	Dong, Meng;Tao, Yanqiang;Wu, Shanshan;Li, Zhengtao;Wang, Xiaobin;Tan, Jichun	2021
165	Single and Multiple Orgasm Experience Among Women in Heterosexual Partnerships. Results of the German Health and Sexuality Survey (GeSiD)	Cerwenka S.; Dekker A.; Pietras L.; Briken P.	2021
166	The impact of the coronavirus disease-19 pandemic on sexual behavior of marriage people in Indonesia	Tribowo J.A.; Tanojo T.D.; Supardi S.; Pakpahan C.; Siswidiyanto E.B.; Rezano A.; I'tishom R.	2021
167	The Impact of the COVID-19 Pandemic on Sexual Behaviors: Findings From a National Survey in the United States	Gleason N.; Banik S.; Braverman J.; Coleman E.	2021
168	Sexual and psychological health of couples with azoospermia in the context of the COVID-19 pandemic	Dong M.; Tao Y.; Wu S.; Li Z.; Wang X.; Tan J.	2021
169	Redefining a sexual medicine paradigm: subclinical premature ejaculation as a new taxonomic entity	Colonnello E.; Ciocca G.; Limoncin E.; Sansone A.; Jannini E.A.	2021
170	The Big "O": Sociocultural Influences on Orgasm Frequency and Sexual Satisfaction in Women	Lentz A.M.; Zaikman Y.	2021
171	Cybersex addiction in a gay man: a case report	Skryabin V.; Zastrozhin M.; Chumakov E.	2021

172	Partnership status, living arrangements, and changes in sexual behaviour and satisfaction during the COVID-19 lockdown: Insights from an observational, cross-sectional online survey in Singapore	Tan R.K.J.; O'Hara C.A.; Kumar N.	2021
173	The disruptive impact of the COVID-19 pandemic on sexual behavior of a German-speaking population; [L'impact de la pandémie de COVID-19 sur l'activité sexuelle et les pratiques sexuelles des célibataires et des personnes en couple dans une population germanophone]	Hille Z.; Oezdemir U.C.; Beier K.M.; Hatzler L.	2021
174	The clinical significance of abnormal ejaculation by silodosin. Is it important?	Akgül M.; Yazıcı C.; Şipal T.; Arda E.	2021
175	Interventions used by allied health professionals in sexual rehabilitation after stroke: A systematic review	Auger L.-P.; Grondin M.; Aubertin M.; Marois A.; Filiatrault J.; Rochette A.	2021
176	The Influence of Types of Stimulation and Attitudes to Clitoral Self-stimulation on Female Sexual and Orgasm Satisfaction: a Cross-sectional Study	Hoy M.; van Stein K.; Strauss B.; Brenk-Franz K.	2021
177	Pornography and Sexual Dissatisfaction: The Role of Pornographic Arousal, Upward Pornographic Comparisons, and Preference for Pornographic Masturbation	Wright P.J.; Paul B.; Herbenick D.; Tokunaga R.S.	2021
178	Pornography Use and Sexual Health among Same-Sex and Mixed-Sex Couples: An Event-Level Dyadic Analysis	Vaillancourt-Morel M.-P.; Rosen N.O.; Štulhofer A.; Bosisio M.; Bergeron S.	2021
179	Exploring Relationships Between Genito-Pelvic Pain/Penetration Disorder, Sex Guilt, and Religiosity Among College Women in the U.S.	Azim K.A.; Happel-Parkins A.; Moses A.; Haardoerfer R.	2021
180	The Impact of Social Distancing Measures Due to COVID-19 Pandemic on Sexual Function and Relationship Quality of Couples in Greece	Sotiropoulou P.; Ferenidou F.; Owens D.; Kokka I.; Minopoulou E.; Koumantanou E.; Pavlopoulou I.; Apotsos P.; Karvouni M.; Koumantarou E.; Mourikis I.; Vaidakis N.; Papageorgiou C.	2021

181	Sexual activity in adolescents and young adults through covid-19 pandemic	Stavridou A.; Samiakou C.; Kourti A.; Tsiorou S.; Panagouli E.; Thirios A.; Psaltopoulou T.; Sergentanis T.N.; Tsitsika A.	2021
182	Sexual quality of life in men <60 years old after coronary bypass surgery	Ghazy T.; Haeberle E.J.; Kappert U.; Petzold S.; Plötze K.; Mashhour A.; Matschke K.; Ouda A.	2021
183	Developing a prediction model for the self-evaluation of erectile dysfunction in an adult male population	Cai Z.; Li H.	2021
184	Uncovering the Most Important Factors for Predicting Sexual Desire Using Explainable Machine Learning	Vowels L.M.; Vowels M.J.; Mark K.P.	2021
185	Penile ring entrapment and strangulation: A case report at Kampala International University Teaching Hospital in Western Uganda	Kyomukama L.A.; Ssebuufu R.; Wani S.A.; Waziri M.A.; Lule H.	2021
186	How the COVID-19 Pandemic Affects Sexual Behavior of Hetero-, Homo-, and Bisexual Males in Germany	Mumm J.-N.; Vilsmaier T.; Schuetz J.M.; Rodler S.; Zati Zehni A.; Bauer R.M.; Staehler M.; Stief C.G.; Batz F.	2021
187	Impact of COVID-19 Pandemic on the Sexual Function of Health Professionals From an Epicenter in Brazil	Neto R.P.; Nascimento B.C.G.; Carvalho dos Anjos Silva G.; Barbosa J.A.B.A.; Júnior J.D.B.; Teixeira T.A.; Srougi M.; Nahas W.C.; Hallak J.; Cury J.	2021
188	Sexual Satisfaction and Frequency of Orgasm in Women With Chronic Pelvic Pain due to Endometriosis	Hämmerli, S; Kohl-Schwartz, A; Imesch, P; Rauchfuss, M; Wölfler, MM; Häberlin, F; von Orelli, S; Leeners, B	2020
189	Disparate Relationship of Sexual Satisfaction, Self-Esteem, Anxiety, and Depression with Endocrine Profiles of Women With or Without PCOS	Glowinska, A; Duleba, AJ; Zielona-Jenek, M; Siakowska, M; Pawelczyk, L; Banaszewska, B	2020
190	Exploratory study of erotic abilities in women's sexual practice	Edard, A; Rusinek, S	2020
191	Picking up good vibrations: Discrepant vibrator use, sexual functioning, and sexual well-being in women with male partners	Gauvin, SEM; Yessick, L; Pukall, CF	2020
192	Assessment of long-term outcomes with immediate versus delayed surgical repair of penile fractures	Haque, MF; Paul, AS; Swain, S; Goyal, G	2020

193	Impact of the COVID-19 Pandemic on Partner Relationships and Sexual and Reproductive Health: Cross-Sectional, Online Survey Study	Li, GJ; Tang, DD; Song, B; Wang, C; Shen, QS; Xu, C; Geng, H; Wu, H; He, XJ; Cao, YX	2020
194	Sexual desire and emotional reactivity in chronically hospitalized Lebanese patients with schizophrenia	Kazour, F; Obeid, S; Hallit, S	2020
195	Pornography Consumption, Modality and Function in a Large Internet Sample	Solano, I; Eaton, NR; O'Leary, KD	2020
196	Does Low-Cost Sexual Gratification Make Men Less Eager to Marry? Pornography Use, Masturbation, Hookup Sex, and Desire to Be Married Among Single Men	Perry, SL	2020
197	Body Image, Orgasmic Response, and Sexual Relationship Satisfaction: Understanding Relationships and Establishing Typologies Based on Body Image Satisfaction	Horvath, Z; Smith, BH; Sal, D; Hevesi, K; Rowland, DL	2020
198	Sexual Expression and Cognitive Function: Gender-Divergent Associations in Older Adults	Wright, H; Jenks, RA; Lee, DM	2020
199	Sexual satisfaction and frequency of orgasm in women with chronic pelvic pain due to endometriosis	Hämmerli, Silvan;Kohl-Schwartz, Alexandra;Imesch, Patrick;Rauchfuss, Martina;Wölfler, Monika M.;Häberlin, Felix;von Orelli, Stephanie;Leeners, Brigitte	2020
200	Sexual Satisfaction and Frequency of Orgasm in Women With Chronic Pelvic Pain due to Endometriosis.	Hämmerli, Silvan;Kohl-Schwartz, Alexandra;Imesch, Patrick;Rauchfuss, Martina;Wölfler, Monika M;Häberlin, Felix;von Orelli, Stephanie;Leeners, Brigitte	2020
201	Body Image, Orgasmic Response, and Sexual Relationship Satisfaction: Understanding Relationships and Establishing Typologies Based on Body Image Satisfaction.	Horvath, Zsolt;Smith, Betina Hodt;Sal, Dorottya;Hevesi, Krisztina;Rowland, David L	2020
202	Does Low-Cost Sexual Gratification Make Men Less Eager to Marry? Pornography Use, Masturbation, Hookup Sex, and Desire to Be Married Among Single Men	Perry, Samuel L	2020

203	Does low-cost sexual gratification make men less eager to marry? Pornography use, masturbation, hookup sex, and desire to be married among single men	Perry, Samuel L.	2020
204	Does Low-Cost Sexual Gratification Make Men Less Eager to Marry? Pornography Use, Masturbation, Hookup Sex, and Desire to Be Married Among Single Men.	Perry, Samuel L	2020
205	Étude exploratoire des habiletés érotiques en jeu dans la pratique sexuelle des femmes	Edard, A.;Rusinek, S.	2020
206	Sexual function, sexual dysfunctions, and ADHD: A systematic literature review	Soldati, Lorenzo;Bianchi-Demicheli, Francesco;Schockaert, Pauline;Köhl, John;Bolmont, Mylène;Hasler, Roland;Perroud, Nader	2020
207	Sexual Function, Sexual Dysfunctions, and ADHD: A Systematic Literature Review.	Soldati, Lorenzo;Bianchi-Demicheli, Francesco;Schockaert, Pauline;Köhl, John;Bolmont, Mylène;Hasler, Roland;Perroud, Nader	2020
208	Impact of the COVID-19 Pandemic on Partner Relationships and Sexual and Reproductive Health: Cross-Sectional, Online Survey Study.	Li, Guanjian;Tang, Dongdong;Song, Bing;Wang, Chao;Qunshan, Shen;Xu, Chuan;Geng, Hao;Wu, Huan;He, Xiaojin;Cao, Yunxia	2020
209	Impact of the COVID-19 Pandemic on Partner Relationships and Sexual and Reproductive Health: Cross-Sectional, Online Survey Study	Li, Guanjian;Tang, Dongdong;Song, Bing;Wang, Chao;Shen Qunshan;Xu, Chuan;Geng, Hao;Wu, Huan;He, Xiaojin;Cao, Yunxia	2020
210	Sexual expression and cognitive function: Gender-divergent associations in older adults	Wright, Hayley;Jenks, Rebecca A.;Lee, David M.	2020
211	Sexual Expression and Cognitive Function: Gender-Divergent Associations in Older Adults.	Wright, Hayley;Jenks, Rebecca A;Lee, David M	2020
212	Sexual Expression and Cognitive Function: Gender-Divergent Associations in Older Adults	Wright, Hayley;Jenks, Rebecca A;Lee, David M	2020
213	Changes in sexual experiences and sexual satisfaction during pregnancy: data from a Greek secondary hospital	Zacharis Konstantinos;Chrysafopoulou Eleni;Kravvaritis Stavros;Charitos Theodoros;Fouka Anastasia	2020

214	Disparate Relationship of Sexual Satisfaction, Self-Esteem, Anxiety, and Depression with Endocrine Profiles of Women With or Without PCOS.	Glowinska, Aleksandra;Duleba, Antoni J;Zielona-Jenek, Monika;Siakowska, Martyna;Pawelczyk, Leszek;Banaszewska, Beata	2020
215	Sexuality, embodiment and attachment style in anorexia nervosa	Cassioli E.; Rossi E.; Castellini G.; Sensi C.; Mancini M.; Lelli L.; Monteleone A.M.; Ricca V.; Stanghellini G.	2020
216	Does Low-Cost Sexual Gratification Make Men Less Eager to Marry? Pornography Use, Masturbation, Hookup Sex, and Desire to Be Married Among Single Men	Perry S.L.	2020
217	Genital Pain and Sexual Functioning: Effects on Sexual Experience, Psychological Health, and Quality of Life	Nimbi F.M.; Rossi V.; Tripodi F.; Luria M.; Flinchum M.; Tambelli R.; Simonelli C.	2020
218	Sexual Satisfaction and Frequency of Orgasm in Women With Chronic Pelvic Pain due to Endometriosis	Hämmerli S.; Kohl-Schwartz A.; Imesch P.; Rauchfuss M.; Wölfler M.M.; Häberlin F.; von Orelli S.; Leeners B.	2020
219	Effects of pornography use and demographic parameters on sexual response during masturbation and partnered sex in women	McNabney S.M.; Hevesi K.; Rowland D.L.	2020
220	Sexual desire and emotional reactivity in chronically hospitalized Lebanese patients with schizophrenia	Kazour F.; Obeid S.; Hallit S.	2020
221	Assessment of long-term outcomes with immediate versus delayed surgical repair of penile fractures	Haque M.; Paul A.; Swain S.; Goyal G.	2020
222	Exploratory study of erotic abilities in women's sexual practice; [Étude exploratoire des habiletés érotiques en jeu dans la pratique sexuelle des femmes]	Edard A.; Rusinek S.	2020
223	Sexual Experiences of Young Transgender Persons During and After Gender-Affirmative Treatment	Bungener S.L.; De Vries A.L.C.; Popma A.; Steensma T.D.	2020
224	Counselled Patients with Stroke Still Experience Sexual and Relational Problems 1–5 Years After Stroke Rehabilitation	Meesters J.J.L.; van de Ven D.P.H.W.; Kruijver E.; Bender J.; Volker W.G.; Vliet Vlieland T.P.M.; Goossens P.H.	2020
225	Sexual Expression and Cognitive Function: Gender-Divergent Associations in Older Adults	Wright H.; Jenks R.A.; Lee D.M.	2020

226	Disparate Relationship of Sexual Satisfaction, Self-Esteem, Anxiety, and Depression with Endocrine Profiles of Women With or Without PCOS	Glowinska A.; Duleba A.J.; Zielona-Jenek M.; Siakowska M.; Pawelczyk L.; Banaszewska B.	2020
227	Body Image, Orgasmic Response, and Sexual Relationship Satisfaction: Understanding Relationships and Establishing Typologies Based on Body Image Satisfaction	Horvath Z.; Smith B.H.; Sal D.; Hevesi K.; Rowland D.L.	2020
228	Self-reported reasons for having difficulty reaching orgasm during partnered sex: relation to orgasmic pleasure	Hevesi K.; Gergely Hevesi B.; Kolba T.N.; Rowland D.L.	2020
229	Relationship Between Masturbation and Partnered Sex in Women: Does the Former Facilitate, Inhibit, or Not Affect the Latter?	Rowland D.L.; Hevesi K.; Conway G.R.; Kolba T.N.	2020
230	Medicalized Pleasure and Silenced Desire: Sexuality of People with Physical Disabilities	Sakairi E.	2020
231	Impact of the COVID-19 pandemic on partner relationships and sexual and reproductive health: Cross-sectional, online survey study	Li G.; Tang D.; Song B.; Wang C.; Qunshan S.; Xu C.; Geng H.; Wu H.; He X.; Cao Y.	2020
232	Body Image Development and Sexual Satisfaction: A Prospective Study From Adolescence to Adulthood	Kvalem, IL; Træen, B; Markovic, A; von Soest, T	2019
233	Sexual Activity and Sexual Satisfaction Among Older Adults in Four European Countries	Træen, B; Stulhofer, A; Janssen, E; Carvalheira, AA; Hald, GM; Lange, T; Graham, C	2019
234	Sexual activity and life satisfaction in older adults	Skalacka, K; Gerymski, R	2019
235	Pornography, preference for porn-like sex, masturbation, and men's sexual and relationship satisfaction	Miller, DJ; McBain, KA; Li, WW; Raggatt, PTF	2019
236	Relationship of Specific Sexual Activities to Orgasmic Latency, Pleasure, and Difficulty During Partnered Sex	Rowland, DL; Kolba, TN	2019
237	Sexuality Among Adults with Congenital Deafblindness: A Cross-Sectional Survey Study Among Primary Carers	Dammeyer, J; Lehane, CM; Chapman, M	2019

238	Sexual Well-Being in Older Men and Women: Construction and Validation of a Multi-Dimensional Measure in Four European Countries	Stulhofer, A; Jurin, T; Graham, C; Enzlin, P; Træen, B	2019
239	Sexual Experiences and Sexual Orientation among Transgender - Adolescents	Stübler, ML; Becker-Hebly, I	2019
240	Contextualizing use of sexual media: Just the first step	Ley, David J.	2019
241	Porn is for masturbation	Prause, Nicole	2019
242	Where does masturbation fit in all this? We need to incorporate measures of solo-masturbation in models connecting sexual media use to sexual quality (or anything else)	Perry, Samuel L.	2019
243	Sexual well-being in older men and women: Construction and validation of a multi-dimensional measure in four European countries	Štulhofer, Aleksandar;Jurin, Tanja;Graham, Cynthia;Enzlin, Paul;Træen, Bente	2019
244	Sexual Well-Being in Older Men and Women: Construction and Validation of a Multi-Dimensional Measure in Four European Countries	Štulhofer, Aleksandar;Jurin, Tanja;Graham, Cynthia;Enzlin, Paul;Træen, Bente	2019
245	Sexuality among adults with congenital deafblindness: A cross-sectional survey study among primary carers	Dammeyer, Jesper;Lehane, Christine M.;Chapman, Madeleine	2019
246	Sexuality Among Adults with Congenital Deafblindness: A Cross-Sectional Survey Study Among Primary Carers	Dammeyer, Jesper;Lehane, Christine M;Chapman, Madeleine	2019
247	Body Image Development and Sexual Satisfaction: A Prospective Study From Adolescence to Adulthood	Kvalem, Ingela Lundin;Træen, Bente;Markovic, Aleksandra;von Soest, Tilmann	2019
248	Body image development and sexual satisfaction: A prospective study from adolescence to adulthood	Kvalem, Ingela Lundin;Træen, Bente;Markovic, Aleksandra;von Soest, Tilmann	2019
249	Body Image Development and Sexual Satisfaction: A Prospective Study From Adolescence to Adulthood.	Kvalem, Ingela Lundin;Træen, Bente;Markovic, Aleksandra;von Soest, Tilmann	2019
250	Frequência sexual e variabilidade da frequência cardíaca numa amostra não-clínica: Resultados preliminares	Gomes, Sabrina;Costa, Rui Miguel;Mangia, Paula;Pestana, José;Coelho, Mafalda Pinto;Correia, Catarina	2019

251	Sexual activity and life satisfaction in older adults	Skałacka, Katarzyna;Gerymski, Rafał	2019
252	Sexual activity and life satisfaction in older adults	Skałacka, Katarzyna;Gerymski, Rafał	2019
253	Sexual activity and life satisfaction in older adults.	Skałacka, Katarzyna;Gerymski, Rafał	2019
254	Relationship of specific sexual activities to orgasmic latency, pleasure, and difficulty during partnered sex	Rowland, David L.;Kolba, Tiffany N.	2019
255	Relationship of Specific Sexual Activities to Orgasmic Latency, Pleasure, and Difficulty During Partnered Sex.	Rowland, David L;Kolba, Tiffany N	2019
256	Sexual activity and sexual satisfaction among older adults in four European countries	Træen, Bente;Štulhofer, Aleksandar;Janssen, Erick;Carvalheira, Ana Alexandra;Hald, Gert Martin;Lange, Theis;Graham, Cynthia	2019
257	Sexual Activity and Sexual Satisfaction Among Older Adults in Four European Countries.	Træen, Bente;Štulhofer, Aleksandar;Janssen, Erick;Carvalheira, Ana Alexandra;Hald, Gert Martin;Lange, Theis;Graham, Cynthia	2019
258	Sexual Activity and Sexual Satisfaction Among Older Adults in Four European Countries	Træen, Bente;Štulhofer, Aleksandar;Janssen, Erick;Carvalheira, Ana Alexandra;Gert Martin Hald;Lange, Theis;Graham, Cynthia	2019
259	Pornography, preference for porn-like sex, masturbation, and men's sexual and relationship satisfaction	Miller, Dan J.;McBain, Kerry A.;Li, Wendy W.;Raggatt, Peter T. F.	2019
260	Sexuelle Erfahrungen und sexuelle Orientierung von Transgender-Jugendlichen	Stübler, Marie Louise;Becker-Hebly, Inga	2019
261	Sexuelle Erfahrungen und sexuelle Orientierung von Transgender-Jugendlichen	Stübler, Marie Louise;Becker-Hebly, Inga	2019
262	Pornography, preference for porn-like sex, masturbation, and men's sexual and relationship satisfaction	Miller, Dan J;McBain, Kerry A;Li, Wendy W;Raggatt, Peter T F	2019
263	THE POINT OF NO RETURN	Pinkerton, Nick	2019
264	Relationship of Specific Sexual Activities to Orgasmic Latency, Pleasure, and Difficulty During Partnered Sex	Rowland D.L.; Kolba T.N.	2019

265	Predictive Factors of Male Sexual Dysfunction after Traumatic Spinal Cord Injury	Ferro J.K.D.O.; Lemos A.; Silva C.P.D.; Lima C.R.O.D.P.; Raposo M.C.F.; Cavalcanti G.D.A.; Oliveira D.A.D.	2019
266	Sexual Activity and Sexual Satisfaction Among Older Adults in Four European Countries	Træen B.; Štulhofer A.; Janssen E.; Carvalheira A.A.; Hald G.M.; Lange T.; Graham C.	2019
267	Sexual frequency and heart rate variability in a portuguese non-clinical sample: Preliminary results; [Frequência sexual e variabilidade da frequência cardíaca numa amostra não-clínica: Resultados preliminares]	Gomes S.; Costa R.M.; Mangia P.; Pestana J.; Coelho M.P.; Correia C.	2019
268	The Role of Sadomasochism in Shaping Violent Sexual Motivation	Semerikova A.A.; Novgorodtsev V.M.; Efimenko D.B.; Melnikova T.E.	2019
269	Body Image Development and Sexual Satisfaction: A Prospective Study From Adolescence to Adulthood	Kvalem I.L.; Træen B.; Markovic A.; von Soest T.	2019
270	Sexuality Among Adults with Congenital Deafblindness: A Cross-Sectional Survey Study Among Primary Carers	Dammeyer J.; Lehane C.M.; Chapman M.	2019
271	Sexual Well-Being in Older Men and Women: Construction and Validation of a Multi-Dimensional Measure in Four European Countries	Štulhofer A.; Jurin T.; Graham C.; Enzlin P.; Træen B.	2019
272	Sexual Experiences and Sexual Orientation among Transgender Adolescents; [Sexuelle Erfahrungen und sexuelle Orientierung von Transgender-Jugendlichen]	Stübler M.L.; Becker-Hebly I.	2019
273	Sexual activity and life satisfaction in older adults	Skałacka K.; Gerymski R.	2019
274	Pornography, preference for porn-like sex, masturbation, and men's sexual and relationship satisfaction	Miller D.J.; McBain K.A.; Li W.W.; Raggatt P.T.F.	2019
275	Retrograde ejaculation associated with quetiapine and treatment with low-dose imipramine	Roughley M.; Lyall M.	2019
276	Sexual satisfaction of patients after the resection of an epileptic focus	Krasuski, T; Jedrzejczak, J; Rysz, A; Owczarek, K	2018

277	Your Vagina Is Not Supposed to Be This Scary Monster: Young Heterosexual Women's Recommendations for Improving Sexual Satisfaction and Implications for Sexuality Education	Blunt-Vinti, HD; Stokowski, SE; Bouza, BM	2018
278	The Relevance of Sexual Concerns and Adult Attachment for Sexual Functioning	Hoy, M; Strauss, B; Brenk-Franz, K	2018
279	Orgasmic Latency and Related Parameters in Women During Partnered and Masturbatory Sex	Rowland, DL; Sullivan, SL; Hevesi, K; Hevesi, B	2018
280	Reasons for Pornography Consumption: Associations with Gender, Psychological and Physical Sexual Satisfaction, and Attitudinal Impacts	Emmers-Sommer, TM	2018
281	Sexual Functioning in a Cohort of Pakistani Men with Spinal Cord Injury	Bin Ayaz, S; Qureshi, AR; Ahmad, A; Gill, ZA; Ahmad, N; Butt, AW	2018
282	Validation of a Visual Analogue Scale to measure the subjective perception of orgasmic intensity in females: The Orgasmometer-F	Mollaioli, D; Di Sante, S; Limoncin, E; Ciocca, G; Gravina, GL; Maseroli, E; Fanni, E; Vignozzi, L; Maggi, M; Lenzi, A; Jannini, EA	2018
283	Sexual functioning in a cohort of Pakistani men with spinal cord injury	Ayaz, Saeed Bin;Qureshi, Ali Raza;Ahmad, Arshia;Gill, Zaheer Ahmed;Ahmad, Nadeem;Butt, Aamir Waheed	2018
284	Sexual Functioning in a Cohort of Pakistani Men with Spinal Cord Injury	Saeed Bin Ayaz;Ali Raza Qureshi;Ahmad, Arshia;Zaheer Ahmed Gill;Ahmad, Nadeem;Aamir Waheed Butt	2018
285	Die Bedeutung von sexuellen Bedenken und Bindung für spezifische sexuelle Funktionsbereiche	Hoy, Madita;Strauß, Bernhard;Brenk-Franz, Katja	2018
286	[The Relevance of Sexual Concerns and Adult Attachment for Sexual Functioning].	Hoy, Madita;Strauß, Bernhard;Brenk-Franz, Katja	2018
287	Orgasmic latency and related parameters in women during partnered and masturbatory sex	Rowland, David L.;Sullivan, Shelbie L.;Hevesi, Krizstina;Hevesi, Barbara	2018
288	Orgasmic Latency and Related Parameters in Women During Partnered and Masturbatory Sex.	Rowland, David L;Sullivan, Shelbie L;Hevesi, Krizstina;Hevesi, Barbara	2018

289	Validation of a Visual Analogue Scale to measure the subjective perception of orgasmic intensity in females: The Orgasmometer-F	Mollaioli, Daniele;Di Sante, Stefania;Limoncin, Erika;Ciocca, Giacomo;Gravina, Giovanni Luca;Maseroli, Elisa;Fanni, Egidia;Vignozzi, Linda;Maggi, Mario;Lenzi, Andrea;Jannini, Emmanuele A.	2018
290	Validation of a Visual Analogue Scale to measure the subjective perception of orgasmic intensity in females: The Orgasmometer-F	Mollaioli, Daniele;Stefania Di Sante;Limoncin, Erika;Ciocca, Giacomo;Gravina, Giovanni Luca;Maseroli, Elisa;Fanni, Egidia;Vignozzi, Linda;Maggi, Mario;Lenzi, Andrea;Emmanuele A Jannini ×	2018
291	[Surgical repair of the tunica albuginea for penis fracture: Selection of incision].	Mao, Yuan-Shen;Hua, Bao;Pan, Wei-Xin;Li, Wen-Feng;Gu, Yu-Fei;Yao, Hai-Jun;Cai, Zhi-Kang;Wang, Zhong;Lu, Chao	2018
292	Reasons for pornography consumption: Associations with gender, psychological and physical sexual satisfaction, and attitudinal impacts	Emmers-Sommer, Tara M.	2018
293	Reasons for Pornography Consumption: Associations with Gender, Psychological and Physical Sexual Satisfaction, and Attitudinal Impacts	Emmers-Sommer, Tara M	2018
294	Validation of a Visual Analogue Scale to measure the subjective perception of orgasmic intensity in females: The Orgasmometer-F.	Mollaioli, Daniele;Di Sante, Stefania;Limoncin, Erika;Ciocca, Giacomo;Gravina, Giovanni Luca;Maseroli, Elisa;Fanni, Egidia;Vignozzi, Linda;Maggi, Mario;Lenzi, Andrea;Jannini, Emmanuele A	2018
295	Bridging exhibitionism and internet pornography: Is there a link?	Chet L.S.; Lin L.J.; Sidi H.; Zakaria H.; Yahaya R.	2018
296	Women's Sexual Health and Aging	Granville L.; Pregler J.	2018
297	Sexuality and Cognitive Status: A U.S. Nationally Representative Study of Home-Dwelling Older Adults	Lindau S.T.; Dale W.; Feldmeth G.; Gavrilova N.; Langa K.M.; Makelarski J.A.; Wroblewski K.	2018
298	Validation of a Visual Analogue Scale to measure the subjective perception of orgasmic intensity in females: The Orgasmometer-F	Mollaioli D.; Sante S.D.; Limoncin E.; Ciocca G.; Gravina G.L.; Maseroli E.; Fanni E.; Vignozzi L.; Maggi M.; Lenzi A.; Jannini E.A.	2018

299	Sexual satisfaction of patients after the resection of an epileptic focus	Krasuski T.; Jędrzejczak J.; Rysz A.; Owczarek K.	2018
300	Chronic periodontal disease correlated with sezual function in young males	Chou M.-H.; Liu C.-Y.; Yang M.-H.; Chou Y.-C.; Wu S.-T.; Cha T.-L.; Tsao C.-W.	2018
301	Sexual Functioning in a Cohort of Pakistani Men with Spinal Cord Injury	Ayaz S.B.; Qureshi A.R.; Ahmad A.; Gill Z.A.; Ahmad N.; Butt A.W.	2018
302	No. 279-Female Sexual Health Consensus Clinical Guidelines	Lamont J.; Bajzak K.; Bouchard C.; Burnett M.; Byers S.; Cohen T.; Fisher W.; Holzapfel S.; Senikas V.	2018
303	Orgasmic Latency and Related Parameters in Women During Partnered and Masturbatory Sex	Rowland D.L.; Sullivan S.L.; Hevesi K.; Hevesi B.	2018
304	Sexual Inhibition is a Vulnerability Factor for Orgasm Problems in Women	Tavares I.M.; Laan E.T.M.; Nobre P.J.	2018
305	Reasons for Pornography Consumption: Associations with Gender, Psychological and Physical Sexual Satisfaction, and Attitudinal Impacts	Emmers-Sommer T.M.	2018
306	Sexual function and quality of life of women adopting the levonorgestrel-releasing intrauterine system (LNG-IUS 13.5 mg) after abortion for unintended pregnancy	Caruso S.; Cianci S.; Vitale S.G.; Fava V.; Cutello S.; Cianci A.	2018
307	The Relevance of Sexual Concerns and Adult Attachment for Sexual Functioning; [Die Bedeutung von sexuellen Bedenken und Bindung für spezifische sexuelle Funktionsbereiche]	Hoy M.; Strau B.; Brenk-Franz K.	2018
308	Dyadic and individual problems of Iranian men with premature ejaculation	Bolghan-Abadi M.; Ahmadi S.A.; Bahrami F.; Fatehizade M.; Jazayeri R.	2018
309	Prevalence of sexual dysfunctions and other sexual disorders in adults with attention-deficit/hyperactivity disorder compared to the general population	Bijlenga D.; Vroege J.A.; Stammen A.J.M.; Breuk M.; Boonstra A.M.; van der Rhee K.; Kooij J.J.S.	2018
310	Satisfaction guaranteed? How individual, partner, and relationship factors impact sexual satisfaction within partnerships	Velten, J; Margraf, J	2017

311	Attachment tendencies and sexual activities: The mediating role of representations of sex	Favez, N; Tissot, H	2017
312	Subjective sleep quality, unstimulated sexual arousal, and sexual frequency	Costa, R; Costa, D; Pestana, J	2017
313	How Do Psychological Risk Factors Predict Sexual Outcomes? A Comparison of Four Models of Young Women's Sexual Outcomes	Tutino, JS; Ouimet, AJ; Shaughnessy, K	2017
314	Sexual Function and Quality of Life Before and After Penile Prosthesis Implantation Following Radial Forearm Flap Phalloplasty	Young, EE; Friedlander, D; Lue, K; Anele, UA; Khurgin, JL; Bivalacqua, TJ; Burnett, AL; Redett, RJ; Gearhart, JP	2017
315	Subjective sleep quality, unstimulated sexual arousal, and sexual frequency.	Costa, Rui;Costa, David;Pestana, José	2017
316	How do psychological risk factors predict sexual outcomes? A comparison of four models of young women’s sexual outcomes	Tutino, Jessica S.;Ouimet, Allison J.;Shaughnessy, Krystelle	2017
317	How Do Psychological Risk Factors Predict Sexual Outcomes? A Comparison of Four Models of Young Women's Sexual Outcomes.	Tutino, Jessica S;Ouimet, Allison J;Shaughnessy, Krystelle	2017
318	Attachment tendencies and sexual activities: The mediating role of representations of sex	Favez, Nicolas;Tissot, Hervé	2017
319	Attachment tendencies and sexual activities: The mediating role of representations of sex	Favez, Nicolas;Tissot, Hervé	2017
320	Sexual Function and Quality of Life Before and After Penile Prosthesis Implantation Following Radial Forearm Flap Phalloplasty.	Young, Ezekiel E;Friedlander, Daniel;Lue, Kathy;Anele, Uzoma A;Khurgin, Jacob L;Bivalacqua, Trinity J;Burnett, Arthur L;Redett, Richard J;Gearhart, John P	2017
321	Satisfaction guaranteed? How individual, partner, and relationship factors impact sexual satisfaction within partnerships	Velten, Julia;Margraf, Jürgen	2017
322	Satisfaction guaranteed? How individual, partner, and relationship factors impact sexual satisfaction within partnerships	Velten, Julia;Margraf, Jürgen	2017

323	Men's sexual and relational adaptations to erectile dysfunction after prostate cancer treatment	Wassersug, Richard J.;Westle, Andrew;Dowsett, Gary W.	2017
324	Satisfaction guaranteed? How individual, partner, and relationship factors impact sexual satisfaction within partnerships.	Velten, Julia;Margraf, Jürgen	2017
325	Men's Sexual and Relational Adaptations to Erectile Dysfunction After Prostate Cancer Treatment	Wassersug, Richard J.;Westle, Andrew;Dowsett, Gary W.	2017
326	The relationship between sexual behavior and internet addiction severity in a sample of heterosexual university students from Italy	Scimeca G.; Muscatello M.R.A.; Chisari C.; Crucitti M.; Pandolfo G.; Zoccali R.; Bruno A.	2017
327	Men's Sexual and Relational Adaptations to Erectile Dysfunction After Prostate Cancer Treatment	Wassersug R.J.; Westle A.; Dowsett G.W.	2017
328	Correlates of Sex Frequency and Sexual Satisfaction Among Partnered Older Adults	Gillespie B.J.	2017
329	Satisfaction guaranteed? How individual, partner, and relationship factors impact sexual satisfaction within partnerships	Velten J.; Margraf J.	2017
330	A Longitudinal Study of Motivations Before and Psychosexual Outcomes After Genital Gender-Confirming Surgery in Transmen	van de Grift T.C.; Pigot G.L.S.; Boudhan S.; Elfering L.; Kreukels B.P.C.; Gijs L.A.C.L.; Buncamper M.E.; Özer M.; van der Sluis W.; Meuleman E.J.H.; Bouman M.-B.; Mullender M.G.	2017
331	Sexual function in young women with type 1 diabetes: the METRO study	Maiorino M.I.; Bellastella G.; Castaldo F.; Petrizzo M.; Giugliano D.; Esposito K.	2017
332	Attachment tendencies and sexual activities: The mediating role of representations of sex	Favez N.; Tissot H.	2017
333	Subjective sleep quality, unstimulated sexual arousal, and sexual frequency	Costa R.; Costa D.; Pestana J.	2017
334	Sexual dysfunction in diabetic patients - An important and overlooked complication	Piątkiewicz P.; Krasuski T.; Maksymiuk-Kłos A.; Owczarek K.	2017

335	How Do Psychological Risk Factors Predict Sexual Outcomes? A Comparison of Four Models of Young Women's Sexual Outcomes	Tutino J.S.; Ouimet A.J.; Shaughnessy K.	2017
336	Sexual Function and Quality of Life Before and After Penile Prosthesis Implantation Following Radial Forearm Flap Phalloplasty	Young E.E.; Friedlander D.; Lue K.; Anele U.A.; Khurgin J.L.; Bivalacqua T.J.; Burnett A.L.; Redett R.J.; Gearhart J.P.	2017
337	Female Sexual Dysfunction—Medical and Psychological Treatments, Committee 14	Kingsberg S.A.; Althof S.; Simon J.A.; Bradford A.; Bitzer J.; Carvalho J.; Flynn K.E.; Nappi R.E.; Reese J.B.; Rezaee R.L.; Schover L.; Shifrin J.L.	2017
338	Implicit and Explicit Sexual Attitudes: How Are They Related to Sexual Desire and Sexual Satisfaction in Men and Women?	Dosch, A; Belayachi, S; Van der Linden, M	2016
339	Sexual well-being of older men and women	Santos-Iglesias, P; Byers, ES; Moglia, R	2016
340	Personality traits, gender roles and sexual behaviours of young adult males	Kurpisz, J; Mak, M; Lew-Starowicz, M; Nowosielski, K; Bienkowski, P; Kowalczyk, R; Misiak, B; Frydecka, D; Samochowiec, J	2016
341	Psychological Factors Involved in Sexual Desire, Sexual Activity, and Sexual Satisfaction: A Multi-factorial Perspective	Dosch, A; Rochat, L; Ghisletta, P; Favez, N; Van der Linden, M	2016
342	Clitorally Stimulated Orgasms Are Associated With Better Control of Sexual Desire, and Not Associated With Depression or Anxiety, Compared With Vaginally Stimulated Orgasms	Prause, N; Kuang, L; Lee, P; Miller, G	2016
343	Male masturbation habits and sexual dysfunctions	Porto, R	2016
344	Habitudes masturbatoires et dysfonctions sexuelles masculines	Porto, R.	2016
345	Clitorally stimulated orgasms are associated with better control of sexual desire, and not associated with depression or anxiety, compared with vaginally stimulated orgasms	Prause, Nicole;Kuang, Lambert;Lee, Peter;Miller, Geoffrey	2016
346	Clitorally Stimulated Orgasms Are Associated With Better Control of Sexual Desire, and Not Associated With Depression or Anxiety, Compared With Vaginally Stimulated Orgasms.	Prause, Nicole;Kuang, Lambert;Lee, Peter;Miller, Geoffrey	2016

347	Psychological Factors Involved in Sexual Desire, Sexual Activity, and Sexual Satisfaction: A Multi-factorial Perspective.	Dosch, Alessandra;Rochat, Lucien;Ghisletta, Paolo;Favez, Nicolas;Van der Linden, Martial	2016
348	Psychological Factors Involved in Sexual Desire, Sexual Activity, and Sexual Satisfaction: A Multi-factorial Perspective	Dosch, Alessandra;Rochat, Lucien;Ghisletta, Paolo;Favez, Nicolas;Van der Linden, Martial	2016
349	Psychological factors involved in sexual desire, sexual activity, and sexual satisfaction: A multi-factorial perspective	Dosch, Alessandra;Rochat, Lucien;Ghisletta, Paolo;Favez, Nicolas;Linden, Martial	2016
350	Personality traits, gender roles and sexual behaviours of young adult males	Kurpisz, Jacek;Mak, Monika;Lew-Starowicz, Michał;Nowosielski, Krzysztof;Bieńkowski, Przemysław;Kowalczyk, Robert;Misiak, Błażej;Frydecka, Dorota;Samochowiec, Jerzy	2016
351	The impact of ejaculatory dysfunction upon the sufferer and his partner.	Abdo, Carmita Helena Najjar	2016
352	Sexual well-being of older men and women	Santos-Iglesias, Pablo;Byers, E Sandra;Moglia, Ronald	2016
353	Altered states of consciousness are related to higher sexual responsiveness	Costa, Rui M.;Pestana, José;Costa, David;Wittmann, Marc	2016
354	Sexual well-being of older men and women	Santos-Iglesias, Pablo;Byers, E. Sandra;Moglia, Ronald	2016
355	Personality traits, gender roles and sexual behaviours of young adult males	Kurpisz, Jacek;Mak, Monika;Lew-Starowicz, Micha;Nowosielski, Krzysztof;Bienkowski, Przemysaw;Kowalczyk, Robert;Misiak, Bazej;Frydecka, Dorota;Samochowiec, Jerzy	2016
356	Personality traits, gender roles and sexual behaviours of young adult males.	Kurpisz, Jacek;Mak, Monika;Lew-Starowicz, Michał;Nowosielski, Krzysztof;Bieńkowski, Przemysław;Kowalczyk, Robert;Misiak, Błażej;Frydecka, Dorota;Samochowiec, Jerzy	2016
357	Sexual health and well-being among older men and women in England: Findings from the English Longitudinal Study of Ageing	Lee, D.M.; Nazroo, J.; O'Connor, D.B.; Blake, M.; Pendleton, N.	2016

358	Clitorally Stimulated Orgasms Are Associated With Better Control of Sexual Desire, and Not Associated With Depression or Anxiety, Compared With Vaginally Stimulated Orgasms	Prause N.; Kuang L.; Lee P.; Miller G.	2016
359	A rubber tube in the bladder as a complication of autoerotic stimulation of the urethra	Stamatiou K.; Moschouris H.	2016
360	The prevalence of sexual activity, and sexual dysfunction and behaviours in postmenopausal woman in Poland	Czajkowska K.B.; Lew-Starowicz Z.; Szymańska M.	2016
361	Women with Disabilities' Perceptions of Sexuality, Sexual Abuse and Masturbation	Morales E.; Gauthier V.; Edwards G.; Courtois F.	2016
362	Personality traits, gender roles and sexual behaviours of young adult males	Kurpisz J.; Mak M.; Lew-Starowicz M.; Nowosielski K.; Bieńkowski P.; Kowalczyk R.; Misiak B.; Frydecka D.; Samochowiec J.	2016
363	Male attitude towards masturbating: an impediment to infertility evaluation and sperm parameters	Pottinger A.M.; Carroll K.; Mason G.	2016
364	Psychological Factors Involved in Sexual Desire, Sexual Activity, and Sexual Satisfaction: A Multi-factorial Perspective	Dosch A.; Rochat L.; Ghisletta P.; Favez N.; Van der Linden M.	2016
365	Male masturbation device for the treatment of premature ejaculation	Rodríguez J.E.; López A.	2016
366	Sexual activity during Pregnancy	Staruch M.; Kucharczyk A.; Zawadzka K.; Wielgos M.; Szymusik I.	2016
367	Male masturbation habits and sexual dysfunctions; [Habitudes masturbatoires et dysfonctions sexuelles masculines]	Porto R.	2016
368	Which are the male factors associated with female sexual dysfunction (FSD)?	Maseroli E.; Fanni E.; Mannucci E.; Fambrini M.; Jannini E.A.; Maggi M.; Vignozzi L.	2016
369	Sexual Satisfaction, Sexual Compatibility, and Relationship Adjustment in Couples: The Role of Sexual Behaviors, Orgasm, and Men's Discernment of Women's Intercourse Orgasm	Klapilová, K; Brody, S; Krejcová, L; Husárová, B; Binter, J	2015

370	The Relationship Between Sexual Satisfaction and Psychological Health of Prison Inmates: The Moderating Effects of Sexual Abstinence and Gender	Carcedo, RJ; Perlman, D; López, F; Orgaz, MB; Fernández-Rouco, N	2015
371	Sexuality for Women With Spinal Cord Injury	Cramp, JD; Courtois, FJ; Ditor, DS	2015
372	Relationships and sexual expression in later life: a biopsychosocial perspective	DeLamater, J; Koepsel, E	2015
373	Sexual Dysfunctional Beliefs Questionnaire (SDBQ): Translation and Psychometric Properties of the Iranian Version	Abdolmanafi, A; Azadfallah, P; Fata, L; Roosta, M; Peixoto, MM; Nobre, P	2015
374	Sexuality and sexual preferences in adolescents and young adults. First results of the German Sex Survey	Zimmermann, I	2015
375	Treatment of Provoked Vulvodynia in a Swedish cohort using desensitization exercises and cognitive behavioral therapy	Lindström, S; Kvist, LJ	2015
376	Sexual function in adult patients with classic bladder exstrophy: A multicenter study	Park, W; Zwink, N; Rösch, WH; Schmiedeke, E; Stein, R; Schmidt, D; Noeker, M; Jenetzky, E; Reutter, H; Ebert, AK	2015
377	Treatment of Provoked Vulvodynia in a Swedish cohort using desensitization exercises and cognitive behavioral therapy.	Lindström, Suzanne;Kvist, Linda J	2015
378	Sexual Dysfunctional Beliefs Questionnaire (SDBQ): Translation and Psychometric Properties of the Iranian Version.	Abdolmanafi, Atefe;Azadfallah, Parviz;Fata, Ladan;Roosta, Mohsen;Peixoto, Maria Manuela;Nobre, Pedro	2015
379	Sexual dysfunctional beliefs questionnaire (SDBQ): Translation and psychometric properties of the Iranian version	Abdolmanafi, Atefe;Azadfallah, Parviz;Fata, Ladan;Roosta, Mohsen;Peixoto, Maria Manuela;Nobre, Pedro	2015
380	Anatomy of sex: Revision of the new anatomical terms used for the clitoris and the female orgasm by sexologists.	Puppo, Vincenzo;Puppo, Giulia	2015
381	Anatomy of sex: Revision of the new anatomical terms used for the clitoris and the female orgasm by sexologists	Puppo, Vincenzo;Puppo, Giulia	2015
382	Sexual satisfaction, sexual compatibility, and relationship adjustment in couples: The role of sexual behaviors, orgasm, and men's discernment of women's intercourse orgasm	Klapilová, Kateřina;Brody, Stuart;Krejčová, Lucie;Husárová, Barbara;Binter, Jakub	2015

383	Sexual satisfaction, sexual compatibility, and relationship adjustment in couples: the role of sexual behaviors, orgasm, and men's discernment of women's intercourse orgasm.	Klapilová, Kateřina;Brody, Stuart;Krejčová, Lucie;Husárová, Barbara;Binter, Jakub	2015
384	Relationships and sexual expression in later life: a biopsychosocial perspective	DeLamater, John;Koepsel, Erica	2015
385	Treatment of Provoked Vulvodynia in a Swedish cohort using desensitization exercises and cognitive behavioral therapy	Lindstrom, Suzanne;Kvist, Linda J	2015
386	Alternative Sexualformen und Beziehungsqualität—Eine online-gestützte empirische Studie	Zimmermann, Ingo;Gossen, Anastasia	2015
387	Relationships and sexual expression in later life: A biopsychosocial perspective	DeLamater, John;Koepsel, Erica	2015
388	Sexual function in adult patients with classic bladder exstrophy: A multicenter study	Park W.; Zwink N.; Rösch W.H.; Schmiedeke E.; Stein R.; Schmidt D.; Noeker M.; Jenetzky E.; Reutter H.; Ebert A.-K.	2015
389	The man whose fetish object is ejaculate: A case report	Taktak Ş.; Karakuş M.; Eke S.M.	2015
390	The potential role of mindfulness in protecting against sexual insecurities	Dunkley C.R.; Goldsmith K.M.; Gorzalka B.B.	2015
391	Treatment of Provoked Vulvodynia in a Swedish cohort using desensitization exercises and cognitive behavioral therapy	Lindström S.; Kvist L.J.	2015
392	Sexual satisfaction, sexual compatibility, and relationship adjustment in couples: The role of sexual behaviors, orgasm, and men's discernment of women's intercourse orgasm	Klapilová K.; Brody S.; Krejčová L.; Husárová B.; Binter J.	2015
393	A new treatment for premature ejaculation? Case series for a desensitizing masturbation aid	Rodríguez J.E.; López A.	2015
394	The existing therapeutic interventions for orgasmic disorders: Recommendations for culturally competent services, narrative review	Salmani Z.; Zargham-Boroujeni A.; Salehi M.; Killeen T.K.; Merghati-Khoei E.	2015
395	Relationships and sexual expression in later life: a biopsychosocial perspective	DeLamater J.; Koepsel E.	2015

396	Sexual Dysfunctional Beliefs Questionnaire (SDBQ): Translation and Psychometric Properties of the Iranian Version	Abdolmanafi A.; Azadfallah P.; Fata L.; Roosta M.; Peixoto M.M.; Nobre P.	2015
397	Masturbation and orgasm as migraine headache treatment: Report of a case	Uca A.U.; Kozak H.H.	2015
398	Removal of Numerous Vesical Magnetic Beads with a Self-Made Magnetic Sheath	Zeng S.-X.; Li H.-Z.; Zhang Z.-S.; Lu X.; Yu X.-W.; Yang Q.-S.; Ma C.; Xu C.-L.; Sun Y.-H.	2015
399	Sexual Functioning and Behavior of Men with Body Dysmorphic Disorder Concerning Penis Size Compared with Men Anxious about Penis Size and with Controls: A Cohort Study	Veale D.; Miles S.; Read J.; Troglia A.; Wylie K.; Muir G.	2015
400	Sex in Its Daily Relational Context	Dewitte M.; Van Lankveld J.; Vandenberghe S.; Loeys T.	2015
401	Coming to power: women's fake orgasms and best orgasm experiences illuminate the failures of (hetero) sex and the pleasures of connection	Fahs, B	2014
402	Personal factors that contribute to or impair women's ability to achieve orgasm	de Lucena, BB; Abdo, CHN	2014
403	Accuracy of 30-Day Recall for Components of Sexual Function and the Moderating Effects of Gender and Mood	Weinfurt, KP; Lin, L; Dombeck, CB; Broderick, JE; Snyder, DC; Williams, MS; Fawzy, MR; Flynn, KE	2014
404	The Impact of Urinary Incontinence on Sexual Function and Sexual Satisfaction in Women with Spinal Cord Injury	Cramp, J; Courtois, F; Connolly, M; Cosby, J; Ditor, D	2014
405	Trajectories of intimate partnerships, sexual attitudes, desire and satisfaction	Ammar, N; Gauthier, JA; Widmer, ED	2014
406	Women's Masturbation: Experiences of Sexual Empowerment in a Primarily Sex-Positive Sample	Bowman, CP	2014
407	Overall satisfaction, sexual function, and the durability of neophallus dimensions following staged female to male genital gender confirming surgery: the Institute of Urology, London U.K. experience	Garcia, MM; Christopher, NA; De Luca, F; Spilotros, M; Ralph, DJ	2014

408	Coming to power: women's fake orgasms and best orgasm experiences illuminate the failures of (hetero)sex and the pleasures of connection	Fahs, Breanne	2014
409	Personal factors that contribute to or impair women's ability to achieve orgasm	de Lucena B B;Abdo C H N	2014
410	Coming to power: Women's fake orgasms and best orgasm experiences illuminate the failures of (hetero)sex and the pleasures of connection	Fahs, Breanne	2014
411	Women's masturbation: Experiences of sexual empowerment in a primarily sex-positive sample	Bowman, Christin P.	2014
412	Personal factors that contribute to or impair women's ability to achieve orgasm.	de Lucena, B B;Abdo, C H N	2014
413	Personal factors that contribute to or impair women's ability to achieve orgasm	De Lucena, B B;Abdo, C H N	2014
414	Coming to power: women's fake orgasms and best orgasm experiences illuminate the failures of (hetero)sex and the pleasures of connection	Fahs, Breanne	2014
415	Coming to power: women's fake orgasms and best orgasm experiences illuminate the failures of (hetero)sex and the pleasures of connection	Fahs, Breanne	2014
416	Overall satisfaction, sexual function, and the durability of neophallus dimensions following staged female to male genital gender confirming surgery: the Institute of Urology, London U.K. experience.	Garcia, Maurice M;Christopher, Nim A;De Luca, Francesco;Spilotros, Marco;Ralph, David J	2014
417	Accuracy of 30-day recall for components of sexual function and the moderating effects of gender and mood	Weinfurt, Kevin P.;Lin, Li;Dombeck, Carrie B.;Broderick, Joan E.;Snyder, Denise C.;Williams, Megan S.;Fawzy, Maria R.;Flynn, Kathryn E.	2014

418	Accuracy of 30-day recall for components of sexual function and the moderating effects of gender and mood.	Weinfurt, Kevin P;Lin, Li;Dombeck, Carrie B;Broderick, Joan E;Snyder, Denise C;Williams, Megan S;Fawzy, Maria R;Flynn, Kathryn E	2014
419	Coming to power: women's fake orgasms and best orgasm experiences illuminate the failures of (hetero)sex and the pleasures of connection.	Fahs, Breanne	2014
420	Penile strangulation due to plastic bottle neck: A surgical emergency	Agarwal A.A.; Singh K.R.; Kushwaha J.K.; Sonkar A.A.	2014
421	The Impact of Vulvar Lichen Sclerosus on Sexual Dysfunction	Haefner H.K.; Aldrich N.Z.; Dalton V.K.; Gagné H.M.; Marcus S.B.; Patel D.A.; Berger M.B.	2014
422	The role of clitoral anatomy in female to male sex reassignment surgery	Vukadinovic V.; Stojanovic B.; Majstorovic M.; Milosevic A.	2014
423	Coming to power: women's fake orgasms and best orgasm experiences illuminate the failures of (hetero)sex and the pleasures of connection	Fahs B.	2014
424	An evidence-based unified definition of lifelong and acquired premature ejaculation: Report of the second international society for sexual medicine Ad Hoc committee for the definition of premature ejaculation	Serefoglu E.C.; Mcmahon C.G.; Waldinger M.D.; Althof S.E.; Shindel A.; Adaikan G.; Becher E.F.; Dean J.; Giuliano F.; Hellstrom W.J.G.; Giraldi A.; Glina S.; Incrocci L.; Jannini E.; Mccabe M.; Parish S.; Rowland D.; Segraves R.T.; Sharlip I.; Torres L.O.	2014
425	Women’s masturbation: Experiences of sexual empowerment in a primarily sex-positive sample	Bowman C.P.	2014
426	Overall satisfaction, sexual function, and the durability of neophallus dimensions following staged female to male genital gender confirming surgery: The Institute of Urology, London U.K. experience	Garcia M.M.; Christopher N.A.; De Luca F.; Spilotros M.; Ralph D.J.	2014
427	Personal factors that contribute to or impair women's ability to achieve orgasm	De Lucena B.B.; Abdo C.H.N.	2014

428	Chapter 8 Sexuality and Menopause		2014
429	Accuracy of 30-Day Recall for Components of Sexual Function and the Moderating Effects of Gender and Mood	Weinfurt K.P.; Lin L.; Dombeck C.B.; Broderick J.E.; Snyder D.C.; Williams M.S.; Fawzy M.R.; Flynn K.E.	2014
430	Pleasure: Neurobiological conception and Freudian conception; [Le plaisir: conception neurobiologique et conception freudienne]	Chenu A.; Tassin J.-P.	2014
431	Female sexual function and the clitoral complex using pelvic MRI assessment	Vaccaro C.M.; Fellner A.N.; Pauls R.N.	2014
432	Relationship Satisfaction as a Predictor of Treatment Response During Cognitive Behavioral Sex Therapy	Stephenson, KR; Rellini, AH; Meston, CM	2013
433	TESTOSTERONIC GADGETS: THE 'NEW' TECHNOLOGY OF ORGASM APPLIED TO SEX TOYS	Freire, SP	2013
434	The Quality of Sexual Experience in Women Correlates with Post-Orgasmic Prolactin Surges: Results from an Experimental Prototype Study	Leeners, B; Kruger, THC; Brody, S; Schmidlin, S; Naegeli, E; Egli, M	2013
435	FACTORS EFFECTING SEXUAL RESPONSE AND SEXUAL SATISFACTION AMONG ELDERLY PERSONS IN BANGKOK, THAILAND	Permpool, P; Yamarat, K; Areesantichai, C	2013
436	Sexual well-being of a community sample of high-functioning adults on the autism spectrum who have been in a romantic relationship	Byers, ES; Nichols, S; Voyer, SD; Reilly, G	2013
437	Parents' Attitudes on Sexual Education - What and When?	Jankovic, S; Malatestinic, G; Striehl, HB	2013
438	Sister-Sister Incest: Data from an Anonymous Computerized Survey	Stroebel, SS; O'Keefe, SL; Griffee, K; Kuo, SY; Beard, KW; Kommor, MJ	2013
439	Alexithymia, Negative Emotions, and Sexual Behavior in Heterosexual University Students from Italy	Scimeca, G; Bruno, A; Pandolfo, G; Micò, U; Romeo, VM; Abenavoli, E; Schimmenti, A; Zoccali, R; Muscatello, MRA	2013
440	ARSH 3: Reproductive and Sexual Health Knowledge: A Comparison Among Married Male and Female Young Adults (15-24 y)	Nair, MKC; Thankachi, Y; Leena, ML; George, B; Russell, PSS	2013

441	Sexual traumatic events and neurotic disorders picture - sexuality-related and sexuality-unrelated symptoms	Sobanski, JA; Klasa, K; Müldner-Nieckowski, L; Dembinska, E; Rutkowski, K; Cyranka, K	2013
442	Immature psychological defense mechanisms are associated with women's greater desire for and actual engaging in masturbation	Brody, Stuart;Nicholson, Susan	2013
443	Immature psychological defense mechanisms are associated with women's greater desire for and actual engaging in masturbation	Brody, Stuart;Nicholson, Susan	2013
444	Immature psychological defense mechanisms are associated with women's greater desire for and actual engaging in masturbation	Brody, Stuart;Nicholson, Susan	2013
445	ARSH 3: Reproductive and sexual health knowledge: a comparison among married male and female young adults (15-24 y).	Nair, M K C;Thankachi, Yamini;Leena, M L;George, Babu;Russell, Paul Swamidhas Sudhakar	2013
446	Sexual Well-Being of a Community Sample of High-Functioning Adults on the Autism Spectrum Who Have Been in a Romantic Relationship	Byers, E. Sandra;Nichols, Shana;Voyer, Susan D.;Reilly, Georgianna	2013
447	Sexual well-being of a community sample of high-functioning adults on the autism spectrum who have been in a romantic relationship	Byers, E. Sandra;Nichols, Shana;Voyer, Susan D.;Reilly, Georgianna	2013
448	Sexual well-being of a community sample of high-functioning adults on the Autism spectrum who have been in a romantic relationship	Byers, E Sandra;Nichols, Shana;Voyer, Susan D;Reilly, Georgianna	2013
449	Psychosexual needs and sexual behaviors of nursing care home residents	Mroczek, Bożena;Kurpas, Donata;Gronowska, Małgorzata;Kotwas, Artur;Karakiewicz, Beata	2013
450	Sexual well-being of a community sample of high-functioning adults on the autism spectrum who have been in a romantic relationship.	Byers, E Sandra;Nichols, Shana;Voyer, Susan D;Reilly, Georgianna	2013
451	[Sexual traumatic events and neurotic disorders picture--sexuality-related and sexuality-unrelated symptoms].	Sobański, Jerzy A;Klasa, Katarzyna;Müldner-Nieckowski, Łukasz;Dembińska, Edyta;Rutkowski, Krzysztof;Cyranka, Katarzyna	2013

452	Parents' attitudes on sexual education--what and when?	Janković, Suzana;Malatestinić, Giulia;Striehl, Henrietta Bencević	2013
453	TESTOSTERONIC GADGETS: THE 'NEW' TECHNOLOGY OF ORGASM APPLIED TO SEX TOYS	Perez Freire, Silvia	2013
454	Sexual traumatic events and neurotic disorders picture—Sexuality-related and sexuality-unrelated symptoms	Sobański, Jerzy A.;Klasa, Katarzyna;Müldner-Nieckowski, Łukasz;Dembińska, Edyta;Rutkowski, Krzysztof;Cyranka, Katarzyna	2013
455	The impact of brief exposure to sexually explicit video clips on partnered female clitoral self-stimulation, orgasm and sexual satisfaction	Kohut, Taylor;Fisher, William A	2013
456	The impact of brief exposure to sexually explicit video clips on partnered female clitoral self-stimulation, orgasm and sexual satisfaction	Kohut, Taylor;Fisher, William A	2013
457	The impact of brief exposure to sexually explicit video clips on partnered female clitoral self-stimulation, orgasm and sexual satisfaction	Kohut, Taylor;Fisher, William A.	2013
458	The impact of brief exposure to sexually explicit video clips on partnered female clitoral self-stimulation, orgasm and sexual satisfaction	Kohut, Taylor;Fisher, William A	2013
459	Parents' attitudes on sexual education - what and when?	Jankovic, S;Malatestinic, G;Striehl, H Bencevic	2013
460	Alexithymia, Negative Emotions, and Sexual Behavior in Heterosexual University Students from Italy	Scimeca, Giuseppe;Bruno, Antonio;Pandolfo, Gianluca;Mico, Umberto;Romeo, Vincenzo M;Abenavoli, Elisabetta;Schimmenti, Adriano;Zoccali, Rocco;Muscatello, Maria R A	2013
461	Alexithymia, negative emotions, and sexual behavior in heterosexual university students from Italy	Scimeca, Giuseppe;Bruno, Antonio;Pandolfo, Gianluca;Micò, Umberto;Romeo, Vincenzo M.;Abenavoli, Elisabetta;Schimmenti, Adriano;Zoccali, Rocco;Muscatello, Maria R. A.	2013

462	Alexithymia, Negative Emotions, and Sexual Behavior in Heterosexual University Students from Italy	Scimeca, Giuseppe;Bruno, Antonio;Pandolfo, Gianluca;Micò, Umberto;Romeo, Vincenzo M;Abenavoli, Elisabetta;Schimmenti, Adriano;Zoccali, Rocco;Muscatello, Maria R; A	2013
463	Alexithymia, negative emotions, and sexual behavior in heterosexual university students from Italy.	Scimeca, Giuseppe;Bruno, Antonio;Pandolfo, Gianluca;Micò, Umberto;Romeo, Vincenzo M;Abenavoli, Elisabetta;Schimmenti, Adriano;Zoccali, Rocco;Muscatello, Maria R A	2013
464	Psychosexual needs and sexual behaviors of nursing care home residents	Mroczek B.; Kurpas D.; Gronowska M.; Kotwas A.; Karakiewicz B.	2013
465	ARSH 3: Reproductive and sexual health knowledge: a comparison among married male and female young adults (15-24 y).	Nair M.K.; Thankachi Y.; Leena M.L.; George B.; Russell P.S.	2013
466	Knowledge and attitude on sex among medical students of a Malaysian university: A comparison study	Sidi H.; Loh S.F.; Mahadevan R.; Puteh S.E.W.; Musa R.; Wong C.Y.; Hadi A.A.A.; Sa'aid S.H.; Amali Z.; Abidin M.; Das S.; Saharom M.H.; Zakaria H.	2013
467	Increased sexual health after restored genital sensation in male patients with spina bifida or a spinal cord injury: The TOMAX procedure	Overgoor M.L.E.; De Jong T.P.V.M.; Cohen-Kettenis P.T.; Edens M.A.; Kon M.	2013
468	The impact of brief exposure to sexually explicit video clips on partnered female clitoral self-stimulation, orgasm and sexual satisfaction	Kohut T.; Fisher W.A.	2013
469	On the essence of sexuality	Klockars L.	2013
470	Immature psychological defense mechanisms are associated with women's greater desire for and actual engaging in masturbation	Brody S.; Nicholson S.	2013
471	A masculine look at pornography - From fantasy to reality; [Point de vue masculin sur la pornographie : Des fantasmes à la réalité]	Lajeunesse S.-L.; Deslauriers J.-M.	2013

472	Alexithymia, negative emotions, and sexual behavior in heterosexual university students from Italy	Scimeca G.; Bruno A.; Pandolfo G.; Micò U.; Romeo V.M.; Abenavoli E.; Schimmenti A.; Zoccali R.; Muscatello M.R.A.	2013
473	Sexual well-being of a community sample of high-functioning adults on the autism spectrum who have been in a romantic relationship	Byers E.S.; Nichols S.; Voyer S.D.; Reilly G.	2013
474	Women's sexuality: From aging to social representations	Ringa V.; Diter K.; Laborde C.; Bajos N.	2013
475	Sister-sister incest: Data from an anonymous computerized survey	Stroebel S.S.; O'Keefe S.L.; Griffee K.; Kuo S.-Y.; Beard K.W.; Kommor M.J.	2013
476	Perceived Effects of Sexually Explicit Media among Men Who Have Sex with Men and Psychometric Properties of the Pornography Consumption Effects Scale (PCES)	Hald G.M.; Smolenski D.; Rosser B.R.S.	2013
477	Standard Operating Procedures in the Disorders of Orgasm and Ejaculation	Mcmahon C.G.; Jannini E.; Waldinger M.; Rowland D.	2013
478	Sexual traumatic events and neurotic disorders picture - Sexuality-related and sexuality-unrelated symptoms; [Seksualne wydarzenia urazowe a obraz zaburzeń nerwicowych - Objawy zwiazane i nie zwiazane z seksualnościa]	Sobański J.A.; Klasa K.; Müldner-Nieckowski Ł.; Dembińska E.; Rutkowski K.; Cyranka K.	2013
479	Parents' attitudes on sexual education - What and when?; [Stavovi roditelja o spolnom odgoju - Što i kada]	Janković S.; Malatestinić G.; Striehl H.B.	2013
480	Medical students' sexuality - development and fulfilment of sexual needs	Müldner-Nieckowski, L; Klasa, K; Sobanski, JA; Rutkowski, K; Dembinska, E	2012
481	Sexual Infidelity in China: Prevalence and Gender-Specific Correlates	Zhang, N; Parish, WL; Huang, YY; Pan, SM	2012
482	SEXUAL DYSFUNCTION IN OBSESSIVE COMPULSIVE DISORDER AND PANIC DISORDER	Aksoy, UM; Aksoy, SG; Maner, F; Gokalp, P; Yanik, M	2012
483	Sexual dysfunction in obsessive compulsive disorder and panic disorder.	Aksoy, Umut Mert;Aksoy, Sennur G;Maner, Fulya;Gokalp, Peykan;Yanik, Medaim	2012

484	Sexual Infidelity in China: Prevalence and Gender-Specific Correlates	Zhang, Na;Parish, William L;Huang, Yingying;Pan, Suiming	2012
485	Sexual infidelity in china: Prevalence and gender-specific correlates	Zhang, Na;Parish, William L.;Huang, Yingying;Pan, Suiming	2012
486	Sexual infidelity in China: prevalence and gender-specific correlates.	Zhang, Na;Parish, William L;Huang, Yingying;Pan, Suiming	2012
487	Sexual Infidelity in China: Prevalence and Gender-Specific Correlates	Zhang, Na;Parish, William L;Huang, Yingying;Pan, Suiming	2012
488	Sexual satisfaction, relationship satisfaction, and health are associated with greater frequency of penile–vaginal intercourse	Costa, Rui Miguel;Brody, Stuart	2012
489	Seksualność studentow medycyny—Rozwój i realizacja potrzeb seksualnych	Müldner-Nieckowski, Łukasz;Klasa, Katarzyna;Sobański, Jerzy A.;Rutkowski, Krzysztof;Dembińska, Edyta	2012
490	Sexual dysfunction in obsessive compulsive disorder and panic disorder	Aksoy, Umut Mert;Aksoy, Şennur G.;Maner, Fulya;Gokalp, Peykan;Yanik, Medaim	2012
491	[Medical students' sexuality--development and fulfilment of sexual needs].	Müldner-Nieckowski, Łukasz;Klasa, Katarzyna;Sobański, Jerzy A;Rutkowski, Krzysztof;Dembińska, Edyta	2012
492	Female Sexual Health Consensus Clinical Guidelines	Lamont J.; Bajzak K.; Bouchard C.; Burnett M.; Byers S.; Cohen T.; Fisher W.; Holzapfel S.; Senikas V.	2012
493	Sexual behavior, body image, and partnership in chronic illness: A comparison of huntington's disease and multiple sclerosis	Reininghaus E.; Reininghaus B.; Fitz W.; Hecht K.; Bonelli R.M.	2012
494	Female Sexual Health Consensus Clinical Guidelines	Lamont J.; Bajzak K.; Bouchard C.; Burnett M.; Byers S.; Cohen T.; Fisher W.; Holzapfel S.; Senikas V.	2012
495	Efficacy of Psychosocial Interventions in Men and Women with Sexual Dysfunctions-A Systematic Review of Controlled Clinical Trials: Part 1-The efficacy of psychosocial interventions for male sexual dysfunction Berner and Günzler Psychosocial Interventions in Male Sexual Dysfunction	Berner M.; Günzler C.	2012

496	Sexual infidelity in China: Prevalence and gender-specific correlates	Zhang N.; Parish W.L.; Huang Y.; Pan S.	2012
497	Medical students' sexuality - Development and fulfilment of sexual needs; [Seksualność studentów medycyny - Rozwój i realizacja potrzeb seksualnych]	Müldner-Nieckowski Ł.; Klasa K.; Sobański J.A.; Rutkowski K.; Dembińska E.	2012
498	Sexual dysfunction in obsessive compulsive disorder and panic disorder	Aksoy U.M.; Aksoy S.G.; Maner F.; Gokalp P.; Yanik M.	2012
499	Medical students' sexuality - Beliefs and attitudes; [Seksualność studenťow medycyny - Przekonania i postawy]	Müldner-Nieckowski Ł.; Sobański J.A.; Klasa K.; Dembińska E.; Rutkowski K.	2012
500	Transtheoretical Model-based Postpartum Sexual Health Education Program Improves Women's Sexual Behaviors and Sexual Health	Lee J.-T.; Tsai J.-L.	2012
501	The relationship between same-sex sexual experience, sexual distress, and female sexual dysfunction	Burri A.; Rahman Q.; Santtila P.; Jern P.; Spector T.; Sandnabba K.	2012
502	Efficacy of Psychosocial Interventions in Men and Women With Sexual Dysfunctions-A Systematic Review of Controlled Clinical Trials: Part 2-The Efficacy of Psychosocial Interventions for Female Sexual Dysfunction Günzler and Berner Psychosocial Interventions in Female Sexual Dysfunction	Günzler C.; Berner M.M.	2012
503	Exploring Women's Postpartum Sexuality: Social, Psychological, Relational, and Birth-Related Contextual Factors	Hipp L.E.; Kane Low L.; Van Anders S.M.	2012
504	Simultaneous Penile-Vaginal Intercourse Orgasm is Associated with Satisfaction (Sexual, Life, Partnership, and Mental Health)	Brody, S; Weiss, P	2011
505	Sexual Behavior Predictors of Satisfaction in a Chinese Sample	Tao, P; Brody, S	2011
506	Sexual media use and relational satisfaction in heterosexual couples	Bridges, AJ; Morokoff, PJ	2011
507	The experiences of men living with inhibited ejaculation	Robbins-Cherry, SA; Hayter, M; Wylie, KR; Goldmeier, D	2011

508	The human female orgasm: critical evaluations of proposed psychological sequelae	Prause, N	2011
509	Quality of Life and Sexual Health after Sex Reassignment Surgery in Transsexual Men	Wierckx, K; Van Caenegem, E; Elaut, E; Dedecker, D; Van de Peer, F; Toye, K; Weyers, S; Hoebeke, P; Monstrey, S; De Cuypere, G; T'Sjoen, G	2011
510	Sexual media use and relational satisfaction in heterosexual couples	Bridges, Ana J.;Morokoff, Patricia J.	2011
511	Quality of life and sexual health after sex reassignment surgery in transsexual men	Wierckx, Katrien;Van Caenegem, Eva;Elaut, Els;Dedecker, David;Van de Peer, Fleur;Toye, Kaatje;Weyers, Steven;Hoebeke, Piet;Monstrey, Stan;De Cuypere, Griet;T'Sjoen, Guy	2011
512	Quality of life and sexual health after sex reassignment surgery in transsexual men.	Wierckx, Katrien;Van Caenegem, Eva;Elaut, Els;Dedecker, David;Van de Peer, Fleur;Toye, Kaatje;Weyers, Steven;Hoebeke, Piet;Monstrey, Stan;De Cuypere, Griet;T'Sjoen, Guy	2011
513	The experiences of men living with inhibited ejaculation	Robbins-Cherry, Sally Ann;Hayter, Mark;Wylie, Kevan Richard;Goldmeier, David	2011
514	The experiences of men living with inhibited ejaculation	Robbins-Cherry, Sally Ann;Hayter, Mark;Wylie, Kevan Richard;Goldmeier, David	2011
515	Practices and sexual behaviors of students of the Universidad de Manizales 2010	Castrillkón, Jose Jaime Castaño;Castaño, Jaime;Cañón, Sandra Constanza;Díaz, Sahira Alejandra;Hernandez, Jhony Alexander;Martínez, Yovanny Hoyos;Marín, Felipe López;Arango, Katherina Manchego;Narvaez, Carlos Wilson;Noguera, Verónica;Ortega, Andrea Marisol	2011
516	Sexual behavior predictors of satisfaction in a Chinese sample	Tao, Peng;Brody, Stuart	2011
517	Sexual behavior predictors of satisfaction in a Chinese sample.	Tao, Peng;Brody, Stuart	2011
518	Frequency of sex in married couples: differences between men and women	Moral de la Rubia, Jose	2011

519	Quality of Life and Sexual Health after Sex Reassignment Surgery in Transsexual Men	Wierckx K.; Van Caenegem E.; Elaut E.; Dedecker D.; Van de Peer F.; Toye K.; Weyers S.; Hoebeke P.; Monstrey S.; De Cuypere G.; T'Sjoen G.	2011
520	Female anorgasmy as a health problem; [Anorgasmia femenina como problema de salud]	Bello L.A.F.; Rivero T.S.E.; Alvarez Y.F.	2011
521	How unusual are the contents of paraphilias? paraphilia-associated sexual arousal patterns in a community-based sample of men	Ahlers C.J.; Schaefer G.A.; Mundt I.A.; Roll S.; Englert H.; Willich S.N.; Beier K.M.	2011
522	Primary lifelong delayed ejaculation: Characteristics and response to bupropion	Abdel-Hamid I.A.; Saleh E.-S.	2011
523	Sexual media use and relational satisfaction in heterosexual couples	Bridges A.J.; Morokoff P.J.	2011
524	The experiences of men living with inhibited ejaculation	Robbins-Cherry S.A.; Hayter M.; Wylie K.R.; Goldmeier D.	2011
525	Sexual Behavior Predictors of Satisfaction in a Chinese Sample	Tao P.; Brody S.	2011
526	Case Studies	Jannini E.A.; Porst H.	2011
527	The relationship between sexual functioning and depressive symptomatology in postpartum women: A pilot study	Chivers M.L.; Pittini R.; Grigoriadis S.; Villegas L.; Ross L.E.	2011
528	Sexuality in women with polycystic ovary syndrome: a pilot study	Silva, JSD; da Fonseca, AM; Bagnoli, VR; Cavalcanti, AL; Soares, JM; Baracat, EC	2010
529	Pornography, Sexual Socialization, and Satisfaction Among Young Men	Stulhofer, A; Busko, V; Landripet, I	2010
530	Influences of Mastery, Spousal Support, and Adaptive Coping on Sexual Drive and Satisfaction Among Chinese Gynecologic Cancer Survivors	Tang, CSK; Lai, BPY; Chung, TKH	2010
531	Sexuality During Pregnancy	Pauleta, JR; Pereira, NM; Graça, LM	2010
532	The International Index of Erectile Function: Development of an Adapted Tool for Use in HIV-Positive Men Who Have Sex with Men	Coyne, K; Mandalia, S; McCullough, S; Catalan, J; Noestlinger, C; Colebunders, R; Asboe, D	2010

533	Exploring the Impact of Prostate Cancer on Men's Sexual Well-Being	Letts, C; Tamlyn, K; Byers, ES	2010
534	Sexuality in women with polycystic ovary syndrome: a pilot study.	Silva, Jucilene Sales da Paixão;Fonseca, Angela Maggio da;Bagnoli, Vicente Renato;Cavalcanti, Ana Lucia;Soares, José Maria;Baracat, Edmund Chada	2010
535	Influences of mastery, spousal support, and adaptive coping on sexual drive and satisfaction among Chinese gynecologic cancer survivors	Tang, Catherine So-kum;Lai, Beatrice P. Y.;Chung, Tony K. H.	2010
536	Influences of mastery, spousal support, and adaptive coping on sexual drive and satisfaction among chinese gynecologic cancer survivors.	Tang, Catherine So-Kum;Lai, Beatrice P Y;Chung, Tony K H	2010
537	Influences of Mastery, Spousal Support, and Adaptive Coping on Sexual Drive and Satisfaction Among Chinese Gynecologic Cancer Survivors	Tang, Catherine So-kum;Lai, Beatrice P; Y;Chung, Tony K; H	2010
538	Exploring the impact of prostate cancer on men's sexual well-being	Letts, Cindy;Tamlyn, Karen;Byers, E. Sandra	2010
539	Exploring the Impact of Prostate Cancer on Men's Sexual Well-Being	Letts, Cindy;Tamlyn, Karen;Byers, E Sandra	2010
540	Exploring the Impact of Prostate Cancer on Men's Sexual Well-Being	Letts, Cindy;Tamlyn, Karen;Byers, E Sandra	2010
541	Pornography, sexual socialization, and satisfaction among young men.	Stulhofer, Aleksandar;Busko, Vesna;Landripet, Ivan	2010
542	The International Index of Erectile Function: development of an adapted tool for use in HIV-positive men who have sex with men.	Coyne, Katherine;Mandalia, Sundhiya;McCullough, Sonya;Catalan, Jose;Noestlinger, Christiana;Colebunders, Robert;Asboe, David	2010
543	Pornography, sexual socialization, and satisfaction among young men	Štulhofer, Aleksandar;Buško, Vesna;Landripet, Ivan	2010

544	The International Index of Erectile Function: Development of an adapted tool for use in HIV-positive men who have sex with men	Coyne, Katherine;Mandalia, Sundhiya;McCullough, Sonya;Catalan, Jose;Noestlinger, Christiana;Colebunders, Robert;Asboe, David	2010
545	Pornography, Sexual Socialization, and Satisfaction Among Young Men	Stulhofer, Aleksandar;Busko, Vesna;Landripet, Ivan	2010
546	Exploring the impact of prostate cancer on men's sexual well-being.	Letts, Cindy;Tamlyn, Karen;Byers, E Sandra	2010
547	Sexuality during pregnancy.	Pauleta, Joana Rocha;Pereira, Nuno Monteiro;Graça, Luís Mendes	2010
548	Sexuality during pregnancy	Pauleta, Joana Rocha;Pereira, Nuno Monteiro;Graça, Luís Mendes	2010
549	Comparison of female sexual dysfunctions before and during pregnancy	Ebrahimian A.; Heydari M.; Saberi Zafarghandi M.B.	2010
550	Love among the objectum sexuals	Marsh A.	2010
551	Sexual satisfaction in dementia: Perspectives of patients and spouses	Dourado M.; Finamore C.; Barroso M.F.; Santos R.; Laks J.	2010
552	Erectile dysfunction and correlated factors in Brazilian men aged 18-40 years	Martins F.G.; Abdo C.H.N.	2010
553	Influences of mastery, spousal support, and adaptive coping on sexual drive and satisfaction among chinese gynecologic cancer survivors	Tang C.S.-K.; Lai B.P.Y.; Chung T.K.H.	2010
554	The development of a sensory substitution system for the sexual rehabilitation of men with chronic spinal cord injury	Borisoff J.F.; Elliott S.L.; Hocaloski S.; Birch G.E.	2010
555	Is infertility a risk factor for female sexual dysfunction? A case-control study	Millheiser L.S.; Helmer A.E.; Quintero R.B.; Westphal L.M.; Milki A.A.; Lathi R.B.	2010
556	Pornography, sexual socialization, and satisfaction among young men	Štulhofer A.; Buško V.; Landripet I.	2010

557	Exploring the impact of prostate cancer on men's sexual well-being	Letts C.; Tamlyn K.; Byers E.S.	2010
558	Differences in predictors of sexual satisfaction and in sexual satisfaction between female and male university students in Slovenia	Ziherl S.; Masten R.	2010
559	Women's vibrator use in sexual partnerships: Results from a nationally representative survey in the United States	Herbenick D.; Reece M.; Sanders S.A.; Dodge B.; Ghassemi A.; Fortenberry J.D.	2010
560	Hypoactive sexual desire disorder: comparison of two questionnaires (the brief profile of female sexual function and the health and female sexual dysfunction in primary care) in women with hysterectomy plus bilateral oophorectomy versus normal women; [Trastorno del deseo sexual hipoactivo: comparación de 2 cuestionarios (breve perfil de la función sexual de la mujer y salud y disfunción sexual femenina en atención primaria) en 2 grupos de mujeres (ovariectomizadas e histerectomizadas, y normales)]	López-Olmos J.	2010
561	Sexuality during pregnancy	Pauleta J.R.; Pereira N.M.; Graça L.M.	2010
562	Expanding the test of counterfeit deviance: Are sexual knowledge, experience and needs a factor in the sexualised challenging behaviour of adults with intellectual disability?	Lockhart K.; Guerin S.; Shanahan S.; Coyle K.	2010
563	Women's sexual desire and arousal disorders	Brotto L.A.; Bitzer J.; Laan E.; Leiblum S.; Luria M.	2010
564	The international index of erectile function: Development of an adapted tool for use in HIV-positive men who have sex with men	Coyne K.; Mandalia S.; McCullough S.; Catalan J.; Noestlinger C.; Colebunders R.; Asboe D.	2010
565	Psychological and interpersonal dimensions of sexual function and dysfunction	McCabe M.; Althof S.E.; Assalian P.; Chevret-Measson M.; Leiblum S.R.; Simonelli C.; Wylie K.	2010
566	Satisfaction (Sexual, Life, Relationship, and Mental Health) Is Associated Directly with Penile-Vaginal Intercourse, but Inversely with Other Sexual Behavior Frequencies	Brody, S; Costa, RM	2009

567	Determinants of Sexual Satisfaction in a Sample of German Women	Philippsohn, S; Hartmann, U	2009
568	Factors Affecting Self-Reported Sexuality in Men with Obstructive Sleep Apnea Syndrome	Stannek, T; Hürny, C; Schoch, OD; Bucher, T; Münzer, T	2009
569	Assessment and treatment of sexual dysfunctions in men and women with spinal cord injury	Courtois, F; Charvier, K; Leriche, A; Côté, M; Lemieux, A	2009
570	Sexual difficulties for persons with multiple sclerosis in New South Wales, Australia	Redelman, MJ	2009
571	Factors affecting self-reported sexuality in men with obstructive sleep apnea syndrome.	Stannek, Tobias;Hürny, Christoph;Schoch, Otto D;Bucher, Thomas;Münzer, Thomas	2009
572	Factors affecting self-reported sexuality in men with obstructive sleep apnea syndrome	Stannek, Tobias;Hürny, Christoph;Schoch, Otto D.;Bucher, Thomas;Münzer, Thomas	2009
573	Satisfaction (sexual, life, relationship, and mental health) is associated directly with penile-vaginal intercourse, but inversely with other sexual behavior frequencies.	Brody, Stuart;Costa, Rui Miguel	2009
574	Satisfaction (sexual, life, relationship, and mental health) is associated directly with penile-vaginal intercourse, but inversely with other sexual behavior frequencies	Brody, Stuart;Costa, Rui Miguel	2009
575	Determinants of sexual satisfaction in a sample of German women.	Philippsohn, Susanne;Hartmann, Uwe	2009
576	Determinants of sexual satisfaction in a sample of German women	Philippsohn, Susanne;Hartmann, Uwe	2009
577	Premature ejaculation: Bother and intravaginal ejaculatory latency time in Iran	Zargooshi J.	2009
578	Sexual functioning in transsexuals following hormone therapy and genital surgery: A review	Klein C.; Gorzalka B.B.	2009

579	Longitudinal changes in sexual functioning as women transition through menopause: Results from the study of women's health across the nation	Avis N.E.; Brockwell S.; Randolph J.F.; Shen S.; Cain V.S.; Ory M.; Greendale G.A.	2009
580	Sexual difficulties for persons with multiple sclerosis in New South Wales, Australia	Redelman M.J.	2009
581	Factors affecting self-reported sexuality in men with obstructive sleep apnea syndrome	Stannek T.; Hürny C.; Schoch O.D.; Bucher T.; Münzer T.	2009
582	Prevalence and characteristics of vibrator use by men in the United States	Reece M.; Herbenick D.; Sanders S.A.; Dodge B.; Ghassemi A.; Fortenberry J.D.	2009
583	Long-term results of immediate surgical treatment of penile fracture	Kim M.G.; Yu J.H.; Sung L.H.; Noh C.H.; Chung J.Y.	2009
584	Pilot study of Australian school-based sexual health education: Parents views	MacBeth A.; Weerakoon P.; Sitharthan G.	2009
585	Satisfaction (sexual, life, relationship, and mental health) Is associated directly with penile-vaginal intercourse, but inversely with other sexual behavior frequencies	Brody S.; Costa R.M.	2009
586	Canadian male sexual health council survey to assess prevalence and treatment of premature ejaculation in Canada	Brock G.B.; Bénard F.; Casey R.; Elliott S.L.; Gajewski J.B.; Lee J.C.	2009
587	Sexual function in women: What is normal?	Domoney C.	2009
588	Determinants of sexual satisfaction in a sample of German women	Philippsohn S.; Hartmann U.	2009
589	Discrepancies between sexual desire and sexual activity: Gender differences and associations with relationship satisfaction	Santtila, P; Wager, I; Witting, K; Harlaar, N; Jern, P; Johansson, A; Varjonen, M; Sandnabba, NK	2008
590	The effects of citalopram and fluoxetine on sexual behavior in healthy men: Evidence of delayed ejaculation and unaffected sexual desire. A randomized, placebo-controlled, double-blind, double-dummy, parallel group study	Madeo, B; Bettica, P; Milleri, S; Balestrieri, A; Granata, ARM; Carani, C; Rochira, V	2008

591	Empathic sexual responses in heterosexual women and men	Haning, R. Vernon;O'Keefe, Stephen L.;Beard, Keith W.;Randall, Elizabeth J.;Kommor, Martin J.;Stroebel, Sandra S.	2008
592	Empathic sexual responses in heterosexual women and men	Haning, R Vernon;O'Keefe, Stephen L;Beard, Keith W;Randall, Elizabeth J;Kommor, Martin J;Stroebel, Sandra S	2008
593	The effects of citalopram and fluoxetine on sexual behavior in healthy men: evidence of delayed ejaculation and unaffected sexual desire. A randomized, placebo-controlled, double-blind, double-dummy, parallel group study.	Madeo, Bruno;Bettica, Paolo;Milleri, Stefano;Balestrieri, Antonio;Granata, Antonio R M;Carani, Cesare;Rochira, Vincenzo	2008
594	The effects of citalopram and fluoxetine on sexual behavior in healthy men: Evidence of delayed ejaculation and unaffected sexual desire. A randomized, placebo-controlled, double-blind, double-dummy, parallel group study	Madeo, Bruno;Bettica, Paolo;Milleri, Stefano;Balestrieri, Antonio;Granata, Antonio R. M.;Carani, Cesare;Rochira, Vincenzo	2008
595	Discrepancies between sexual desire and sexual activity: gender differences and associations with relationship satisfaction.	Santtila, Pekka;Wager, Ingrid;Witting, Katarina;Harlaar, Nicole;Jern, Patrick;Johansson, Ada;Varjonen, Markus;Sandnabba, N Kenneth	2008
596	Discrepancies between sexual desire and sexual activity: Gender differences and associations with relationship satisfaction	Santtila, Pekka;Wager, Ingrid;Witting, Katarina;Harlaar, Nicole;Jern, Patrick;Johansson, Ada;Varjonen, Markus;Sandnabba, N. Kenneth	2008
597	Discrepancies between sexual desire and sexual activity: Gender differences and associations with relationship satisfaction	Santtila P.; Wager I.; Witting K.; Harlaar N.; Jern P.; Johansson A.; Varjonen M.; Sandnabba N.K.	2008
598	Effectiveness of sildenafil citrate (Viagra™) and tadalafil (Cialis™) on sexual responses in Saudi men with erectile dysfunction in routine clinical practice	Ali S.T.	2008
599	Clinical holistic medicine: Teaching orgasm for females with chronic anorgasmia using the Betty Dodson method	Struck P.; Ventegodt S.	2008
600	The prevalence and risk factors of female sexual dysfunction in young Korean women: An internet-based survey	Song S.H.; Jeon H.; Kim S.W.; Paick J.-S.; Son H.	2008

601	The effects of citalopram and fluoxetine on sexual behavior in healthy men: Evidence of delayed ejaculation and unaffected sexual desire. A randomized, placebo-controlled, double-blind, double-dummy, parallel group study	Madeo B.; Bettica P.; Milleri S.; Balestrieri A.; Granata A.R.M.; Carani C.; Rochira V.	2008
602	Empathic sexual responses in heterosexual women and men	Haning R.V.; O'Keefe S.L.; Beard K.W.; Randall E.J.; Kommor M.J.; Stroebel S.S.	2008
603	The relationship between serial sexual murder and autoerotic asphyxiation	Myers W.C.; Bukhanovskiy A.; Justen E.; Morton R.J.; Tilley J.; Adams K.; Vandagriff V.L.; Hazelwood R.R.	2008
604	Sexual functioning of people with rheumatoid arthritis: a multicenter study	van Berlo, WTM; van de Wiel, HBM; Taal, E; Rasker, JJ; Schultz, WCMW; van Rijswijk, MH	2007
605	Body Image and Sexual Functioning Among Bisexual Women	Zamboni, BD; Robinson, BE; Bockting, WO	2007
606	Masturbation in the United States	Das, A	2007
607	Masturbation in the United States.	Das, Aniruddha	2007
608	Masturbation in the United States	Das, Aniruddha	2007
609	Vaginal orgasm is associated with better psychological function	Brody, Stuart	2007
610	Vaginal orgasm is associated with better psychological function	Brody, Stuart	2007
611	Sexual activity, health and well-being--the beneficial roles of coitus and masturbation	Levin, Roy J.	2007
612	Sexual activity, health and well-being - the beneficial roles of coitus and masturbation	Levin, Roy J	2007
613	Sexual functioning of people with rheumatoid arthritis: a multicenter study.	van Berlo, Willy T M;van de Wiel, Harry B M;Taal, Erik;Rasker, Johannes J;Weijmar Schultz, Willibrord C M;van Rijswijk, Martin H	2007
614	Sexual functioning of people with rheumatoid arthritis: a multicenter study	Willy T M van Berlo;Harry B M van de Wiel;Taal, Erik;Rasker, Johannes J;Willibrord C M Weijmar Schultz;van Rijswijk, Martin H	2007
615	Body image and sexual functioning among bisexual women	Zamboni, Brian D.;Robinson, Beatrice “Bean” E.;Bockting, Walter O.	2007

616	Sexual Behavior in Later Life	DeLamater, J.; Moorman, S.M.	2007
617	Sexual functioning of people with rheumatoid arthritis: A multicenter study	van Berlo W.T.M.; van de Wiel H.B.M.; Taal E.; Rasker J.J.; Weijmar Schultz W.C.M.; van Rijswijk M.H.	2007
618	Sex after seventy: A pilot study of sexual function in older persons	Smith L.J.; Mulhall J.P.; Deveci S.; Monaghan N.; Reid M.C.	2007
619	Masturbation in the United States	Das A.	2007
620	Vaginal orgasm is associated with better psychological function	Brody S.	2007
621	Premature ejaculation: A new approach by James H. Semans	Glina S.Sidney; Abdo C.H.N.; Waldinger M.D.; Althof S.E.; Mc Mahon C.; Salonia A.; Donatucci C.	2007
622	The racialisation and ethnicisation of sexuality and sexual problems in sex therapeutic discourse	Mulholland J.	2007
623	Sexual Function After Three-Dimensional Conformal Radiotherapy for Prostate Cancer: Results From a Dose-Escalation Trial	van der Wielen G.J.; van Putten W.L.J.; Incrocci L.	2007
624	Combined brief psychosexual intervention after mastectomy: Effects on sexuality, body image, and psychological well-being	Kalaitzi C.; Papadopoulos V.P.; Michas K.; Vlasis K.; Skandalakis P.; Filippou D.	2007
625	French doctors and masturbation before 1945; [Les médecins français et la masturbation avant 1945]	Brenot P.	2007
626	Prevalence and correlates of sexual dysfunction among young adult married women in rural China: a population-based study	Lau, JTF; Cheng, Y; Wang, Q; Yang, X	2006
627	Seminal characteristics and sexual behavior in men of different age groups: is there an aging effect?	Zavos, PM; Kaskar, K; Correa, JR; Sikka, SC	2006
628	[Sexuality of pregnant women].	Malarewicz, Andrzej;Szymkiewicz, Jadwiga;Rogala, Jerzy	2006
629	Prevalence and correlates of sexual dysfunction among young adult married women in rural China: a population-based study.	Lau, J T F;Cheng, Y;Wang, Q;Yang, X	2006
630	Prevalence and correlates of sexual dysfunction among young adult married women in rural China: a population-based study	Lau J T F;Cheng, Y;Wang, Q;Yang, X	2006

631	Prevalence and correlates of sexual dysfunction among young adult married women in rural China: a population-based study	Lau, J T F;Cheng, Y;Wang, Q;Yang, X	2006
632	Prevalence and correlates of sexual dysfunction among young adult married women in rural China: A population-based study	Lau J.T.F.; Cheng Y.; Wang Q.; Yang X.	2006
633	Sexual behaviours among students of the Medical University in Gdańsk; [Formy zachowań seksualnych wśród studentów Akademii Medycznej w Gdańsku]	Grabowski K.; Wichowicz H.M.; Cubała W.J.	2006
634	Premature ejaculation	Broderick G.A.	2006
635	The post-orgasmic prolactin increase following intercourse is greater than following masturbation and suggests greater satiety	Brody S.; Krüger T.H.C.	2006
636	On orgasm, sexual techniques, and erotic perceptions in 18- to 74-year-old Swedish women	Fugl-Meyer K.S.; Öberg K.; Lundberg P.O.; Lewin B.; Fugl-Meyer A.	2006
637	Penile - Vaginal intercourse is better: Evidence trumps ideology	Brody S.	2006
638	A double-blind study of citalopram versus placebo in the treatment of compulsive sexual behaviors in gay and bisexual men	Wainberg M.L.; Muench F.; Morgenstern J.; Hollander E.; Irwin T.W.; Parsons J.T.; Allen A.; O'Leary A.	2006
639	Patient-reported complications and functional outcomes of male-to-female sex reassignment surgery	Lawrence A.A.	2006
640	Sexual function and sexual activities in female schizophrenic patients; [Sexuální funkce a sexuální aktivity pacientek s onemocněním schizofrenního okruhu]	Mansour-Musová H.; Weiss P.	2006
641	Body image and Sexual functioning among bisexual women	Zamboni B.D.; Robinson B.B.E.; Bockting W.O.	2006
642	A genealogy of the genital kiss: Oral sex in the twentieth century	Hunt A.; Curtis B.	2006
643	Sexual dysfunctions and marital adjustment in veterans with PTSD	Ahmadi K.; Fathi-Ashtiani A.; Zareir A.; Arabnia A.; Amiri M.	2006
644	Prevalence and risk factors of sexual dysfunction among younger married men in a rural area in China	Lau, JTF; Wang, QS; Cheng, YM; Yang, XL	2005
645	Persistent sexual arousal syndrome: A descriptive study	Leiblum, S; Brown, C; Wan, J; Rawlinson, L	2005

646	Clitoral therapy device for treatment of sexual dysfunction in irradiated cervical cancer patients	Schroder, M; Mell, LK; Hurteau, JA; Collins, YC; Rotmensch, J; Waggoner, SE; Yamada, SD; Small, W; Mundt, AJ	2005
647	Sexual and physical health after sex reassignment surgery	De Cuypere, G; T'Sjoen, G; Beerten, R; Selvaggi, G; De Sutter, P; Hoebeke, P; Monstrey, S; Vansteenwegen, A; Rubens, R	2005
648	Sexual and physical health after sex reassignment surgery.	De Cuypere, Griet;T'Sjoen, Guy;Beerten, Ruth;Selvaggi, Gennaro;De Sutter, Petra;Hoebeke, Piet;Monstrey, Stan;Vansteenwegen, Alfons;Rubens, Robert	2005
649	Sexual and Physical Health After Sex Reassignment Surgery	De Cuypere, Griet;T'Sjoen, Guy;Beerten, Ruth;Selvaggi, Gennaro;De Sutter, Petra;Hoebeke, Piet;Monstrey, Stan;Vansteenwegen, Alfons;Rubens, Robert	2005
650	Sexual and Physical Health After Sex Reassignment Surgery	De Cuypere Griet;TSjoen Guy;Beerten, Ruth;Selvaggi Gennaro;De Sutter Petra;Hoebeke Piet;Monstrey Stan;Vansteenwegen Alfons;Rubens, Robert	2005
651	Sexual and Physical Health After Sex Reassignment Surgery	De Cuypere, Griet;TSjoen, Guy;Beerten, Ruth;Selvaggi, Gennaro;De Sutter, Petra;Hoebeke, Piet;Monstrey, Stan;Vansteenwegen, Alfons;Rubens, Robert	2005
652	Prevalence and risk factors of sexual dysfunction among younger married men in a rural area in China.	Lau, Joseph T F;Wang, Qingsheng;Cheng, Yimin;Yang, Xilin	2005
653	Persistent sexual arousal syndrome: a descriptive study.	Leiblum, Sandra;Brown, Candace;Wan, Jim;Rawlinson, Leslie	2005
654	Persistent sexual arousal syndrome: A descriptive study	Leiblum, Sandra;Brown, Candace;Wan, Jim;Rawlinson, Leslie	2005
655	Clitoral therapy device for treatment of sexual dysfunction in irradiated cervical cancer patients.	Schroder, Maryann;Mell, Loren K;Hurteau, Jean A;Collins, Yvonne C;Rotmensch, Jacob;Waggoner, Steven E;Yamada, S Diane;Small, William;Mundt, Arno J	2005
656	Douleur et perversions -- Troubles factices -- Syndrome de Münchausen. Pathomimies	Defontaine-Catteau, M. Cl.	2005

657	The incidental orgasm: The presence of clitoral knowledge and the absence of orgasm for women	Wade L.D.; Kremer E.C.; Brown J.	2005
658	Persistent sexual arousal syndrome: A descriptive study	Leiblum S.; Brown C.; Wan J.; Rawlinson L.	2005
659	Prevalence and risk factors of sexual dysfunction among younger married men in a rural area in China	Lau J.T.F.; Wang Q.; Cheng Y.; Yang X.	2005
660	Sexual and physical health after sex reassignment surgery	De Cuypere G.; T'Sjoen G.; Beerten R.; Selvaggi G.; De Sutter P.; Hoebeke P.; Monstrey S.; Vansteenwegen A.; Rubens R.	2005
661	Clitoral therapy device for treatment of sexual dysfunction in irradiated cervical cancer patients	Schroder M.; Mell L.K.; Hurteau J.A.; Collins Y.C.; Rotmensch J.; Waggoner S.E.; Yamada S.D.; Small Jr. W.; Mundt A.J.	2005
662	Predicting sexual satisfaction in women: Implications for counselor education and training	Bridges, SK; Lease, SH; Ellison, CR	2004
663	Religiosity and sexual responsibility: Relationships of choice	Davidson, JK; Moore, NB; Ullstrup, KM	2004
664	Lifetime depression history and sexual function in women at midlife	Cyranowski, JM; Bromberger, J; Youk, A; Matthews, K; Kravitz, HM; Powell, LH	2004
665	Changes in sexual function in middle-aged and older men: Longitudinal data from the Massachusetts Male Aging Study	Araujo, AB; Mohr, BA; McKinlay, JB	2004
666	Lifetime depression history and sexual function in women at midlife.	Cyranowski, Jill M;Bromberger, Joyce;Youk, Ada;Matthews, Karen;Kravitz, Howard M;Powell, Lynda H	2004
667	Lifetime Depression History and Sexual Function in Women at Midlife	Cyranowski, Jill M.;Bromberger, Joyce;Youk, Ada;Matthews, Karen;Kravitz, Howard M.;Powell, Lynda H.	2004
668	Lifetime Depression History and Sexual Function in Women at Midlife	Cyranowski, Jill M;Bromberger, Joyce;Youk, Ada;Matthews, Karen;Kravitz, Howard M;Powell, Lynda H	2004
669	Changes in sexual function in middle-aged and older men: longitudinal data from the Massachusetts Male Aging Study.	Araujo, Andre B;Mohr, Beth A;McKinlay, John B	2004
670	Changes in Sexual Function in Middle-Aged and Older Men: Longitudinal Data from the Massachusetts Male Aging Study	Araujo, Andre B.;Mohr, Beth A.;McKinlay, John B.	2004

671	Changes in sexual function in middle-aged and older men: longitudinal data from the Massachusetts male aging study	Araujo, Andre B;McKinlay, John B;Mohr, Beth A	2004
672	Changes in sexual function in middle-aged and older men: Longitudinal data from the Massachusetts Male Aging Study	Araujo, Andre B;Mohr, Beth A;McKinlay, John B	2004
673	Religiosity and sexual responsibility: relationships of choice.	Davidson, J Kenneth;Moore, Nelwyn B;Ullstrup, Kristen Marie	2004
674	Religiosity and Sexual Responsibility: Relationships of Choice	Davidson, J. Kenneth;Moore, Nelwyn B.;Ullstrup, Kristen Marie	2004
675	Religiosity and Sexual Responsibility: Relationships of Choice	Davidson, J. Kenneth;Moore, Nelwyn B.;Ullstrup, Kristen Marie	2004
676	Religiosity and sexual responsibility: relationships of choice	Davidson, J Kenneth;Moore, Nelwyn B;Ullstrup, Kristen Marie	2004
677	Predicting Sexual Satisfaction in Women: Implications for Counselor Education and Training	Bridges, Sara K.;Lease, Suzanne H.;Ellison, Carol R.	2004
678	Predicting sexual satisfaction in women: implications for counselor education and training	Bridges, Sara K;Ellison, Carol R;Lease, Suzanne H	2004
679	Predicting Sexual Satisfaction in Women: Implications for Counselor Education and Training	Bridges, Sara K;Lease, Suzanne H;Ellison, Carol R	2004
680	Lifetime depression history and sexual function in women at midlife	Cyranowski J.M.; Bromberger J.; Youk A.; Matthews K.; Kravitz H.M.; Powell L.H.	2004
681	Changes in sexual function in middle-aged and older men: Longitudinal data from the Massachusetts male aging study	Araujo A.B.; Mohr B.A.; McKinlay J.B.	2004
682	Religiosity and sexual responsibility: Relationships of choice	Davidson Sr. J.K.; Moore N.B.; Ullstrup K.M.	2004
683	Sexuality in healthy postmenopausal women	Penteado, SRL; Fonseca, AM; Bagnoli, VR; Assis, JS; Pinotti, JA	2003
684	Sexual satisfaction in young adulthood: Cohabitation, committed dating or unattached life?	Pedersen, W.; Blekesaune, M.	2003

685	Sexuality in healthy postmenopausal women.	Penteado, S R L;Fonseca, A M;Bagnoli, V R;Assis, J S;Pinotti, J A	2003
686	Sexuality in healthy postmenopausal women	Penteado, S R L;Fonseca, A M;Bagnoli, V R;Assis, J S;Pinotti, J A	2003
687	Sexual functioning of women with HIV: A comparison with non-HIV women	Denis, Alicia;Hong, Sung-Mook	2003
688	SEXUAL FUNCTIONING OF WOMEN WITH HIV: A COMPARISON WITH NON-HIV WOMEN	Denis, Alicia;Sung-Mook, Hong	2003
689	Sexuelle Befriedigung und Sexualmythen bei Frauen: Ergebnisse einer Fragebogenuntersuchung zu den Determinanten sexueller Zufriedenheit	Philippsohn, Susanne;Heiser, Kristina;Hartmann, Uwe	2003
690	Sexual functioning of women with HIV: a comparison with non-HIV women	Denis, Alicia;Hong, Sung-Mook	2003
691	Sexual functioning of women with HIV: a comparison with non-HIV women	Denis, Alicia;Hong, Sung-Mook	2003
692	Sexuality in healthy postmenopausal women	Penteado S.R.L.; Fonseca A.M.; Bagnoli V.R.; Assis J.S.; Pinotti J.A.	2003
693	Sexual satisfaction and sexual myths among women: Results of a survey on the determinants of sexual satisfaction; [Sexuelle befriedigung und sexualmythen bei frauen: Ergebnisse einer fragebogenuntersuchung zu den determinanten sexueller zufriedenheit]	Philippsohn S.; Heiser K.; Hartmann U.	2003
694	Sexuality following the menopause: The influence of hormone replacement therapy onwomen with sexual dysfunction; [Sexualidad tras la menopausia: Influencia del tratamiento hormonal sustitutivo en las disfunciones de la mujer]	López-Olmos J.	2003
695	Masturbation as a means of achieving sexual health	Coleman E.	2003

696	Sexual functioning of women with HIV: A comparison with non-HIV women	Denis A.; Hong S.-M.	2003
697	Relationship functioning and sexuality among people with multiple sclerosis	McCabe, MP	2002
698	Sexual functioning and self-reported depressive symptoms among college women	Frohlich, P; Meston, C	2002
699	Relationship functioning and sexuality among people with multiple sclerosis.	McCabe, Marita P	2002
700	Sexual functioning and self-reported depressive symptoms among college women.	Frohlich, Penny;Meston, Cindy	2002
701	Relationship functioning and sexuality among people with Multiple Sclerosis	McCabe, Marita P.	2002
702	Sexual functioning and self-reported depressive symptoms among college women	Frohlich, Penny;Meston, Cindy	2002
703	Sexual functioning and self-reported depressive symptoms among college women	Frohlich, Penny;Meston, Cindy	2002
704	Relationship functioning and sexuality among people with multiple sclerosis	McCabe, Marita P	2002
705	Masturbation as a means of achieving sexual health	Coleman, Eli	2002
706	Sexual functioning and self-reported depressive symptoms among college women	Frohlich P.; Meston C.	2002
707	Sexual motivation and the duration of partnership	Klusmann D.	2002
708	On sexual well-being in sexually abused Swedish women: Epidemiological aspects	Öberg K.; Fugl-Meyer K.S.; Fugl-Meyer A.R.	2002
709	Relationship functioning and sexuality among people with multiple sclerosis	McCabe M.P.	2002

710	Sexual problems in men with prostate cancer in comparison with men with benign prostatic hyperplasia and men from the general population	Jakobsson, L; Lovén, L; Hallberg, IR	2001
711	Sexual problems in men with prostate cancer in comparison with men with benign prostatic hyperplasia and men from the general population	Jakobsson, L;Loven, L;Hallberg, I R	2001
712	Etnicidad y género en la comunidad universitaria	Mercedes Fernández B;Sandra Zúñiga B	2001
713	Sade: The Invention of the Libertine Body (review)	Julie Candler Hayes	2001
714	Douleur et perversions	Defontaine-Catteau M.-C.	2001
715	Sexual problems in men with prostate cancer in comparison with men with benign prostatic hyperplasia and men from the general population	Jakobsson L.; Lovén L.; Hallberg I.R.	2001
716	Peyronie's disease: Epidemiology and clinical presentation of 134 cases	Perimenis P.; Athanasopoulos A.; Gyftopoulos K.; Katsenis G.; Barbalias G.	2001
717	La determination du degre de relation entre l'estime de soi et quatre elements de l'experience sexuelle	Belanger, Jean;Piche, Lyne;Trudel, Gilles	2000
718	Role of oestrogen in male sexual behaviour: insights from the natural model of aromatase deficiency	Carani, C; Rochira, V; Faustini-Fustini, M; Balestrieri, A; Granata, ARM	1999
719	Role of oestrogen in male sexual behaviour: insights from the natural model of aromatase deficiency.	Carani, C;Rochira, V;Faustini-Fustini, M;Balestrieri, A;Granata, A R	1999
720	Role of oestrogen in male sexual behaviour: Insights from the natural model of aromatase deficiency	Carani C.; Rochira V.; Faustini-Fustini M.; Balestrieri A.; Granata A.R.M.	1999
721	The long-term safety of alprostadil (prostaglandin-E1) in patients with erectile dysfunction	Alvarez, E; Andrianne, R; Arvis, G; Boezaart, F; Buvat, J; Czyzyk, A; de Boccard, GA; Declercq, G; Di Silverio, F; Drent, D; du Preez, M; Heal, M; Henriet, J; Jardin, A; Leriche, A; Lochner-Ernst, D; Martelli, A; Marx, W; Mattarelli, G; Merckx, L; Meuleman, EJH; Mirone, V; Morgan, J; Naude, JH; Navratil, H; Noren, S; Pagano, F; Pomerol, JM; Porst, H;	1998

		Pryor, J; Sandhu, DPS; Schaefer, RM; Schmidt, A; Stackl, W; Staehler, G; Stein, R; Sulke, J; van Ahlen, H; Vaderschueren, D; Weiske, WH; Wespes, E; Borg, G	
722	The long-term safety of alprostadil (prostaglandin-E1) in patients with erectile dysfunction. The European Alprostadil Study Group.	1998	
723	The long-term safety of alprostadil (prostaglandin-Ej) in patients with erectile dysfunction	Borg G.	1998
724	Effect of testosterone administration on sexual behavior and mood in men with erectile dysfunction	Schiavi, RC; White, D; Mandeli, J; Levine, AC	1997
725	Sexuální aktivita obyvatel České republiky: Výsledky národního výzkumu	Weiss, Petr;Zvěřina, J.	1997
726	Effect of testosterone administration on sexual behavior and mood in men with erectile dysfunction.	Schiavi, R C;White, D;Mandeli, J;Levine, A C	1997
727	Effect of testosterone administration on sexual behavior and mood in men with erectile dysfunction	Schiavi, Raul C.;White, Daniel;Mandeli, John;Levine, Alice C.	1997
728	Effect of testosterone administration on sexual behavior and mood in men with erectile dysfunction	Schiavi, Raul C;White, Daniel;Mandeli, John;Levine, Alice C	1997
729	Effect of testosterone administration on sexual behavior and mood in men with erectile dysfunction	Schiavi R.C.; White D.; Mandeli J.; Levine A.C.	1997
730	Czech women's experience with sexual violence. Results of a national survey; [ZKUSENOSTI CESKYCH ZEN SE SEXUALNIM NASILIM. VYSLEDKY NARODNIHO PRUZKUMU]	Weiss P.; Zverina J.	1997
731	The sexual experience of women diagnosed with anorexia nervosa or bulimia nervosa	Wiederman, MW; Pryor, T; Morgan, CD	1996
732	Clomipramine and sexual function in men with premature ejaculation and controls	Haensel, SM; Rowland, DL; Kallan, KTHK; Slob, AK	1996

733	Clomipramine and sexual function in men with premature ejaculation and controls.	Haensel, S M;Rowland, D L;Kallan, K T	1996
734	The sexual experience of women diagnosed with anorexia nervosa or bulimia nervosa.	Wiederman, M W;Pryor, T;Morgan, C D	1996
735	Sexual behavior of teenagers: Conclusions from the results of a comparative study between two surveys: South Brazil	de Souza, Ronald Pagnoncelli;de Oliveira, José Sperb;Wagner, Mario Bernardes;Vinciprova, Andréia Rosa	1996
736	The sexual experience of women diagnosed with anorexia nervosa or bulimia nervosa	Wiederman, Michael W.;Pryor, Tamara;Morgan, C. Don	1996
737	Erectile dysfunction in men with chronic medical illness	Plaud, Joseph J.;Dubbert, Patricia M.;Holm, Jeffrey;Wittrock, David;Smith, Patrick;Edison, Janis;McAnulty, Richard;Caddell, Juesta;Summerville, Mary;Jones, Adolph	1996
738	Clomipramine and sexual function in men with premature ejaculation and controls	Haensel S.M.; Rowland D.L.; Kallan K.T.H.K.; Slob A.K.	1996
739	The sexual experience of women diagnosed with anorexia nervosa or bulimia nervosa	Wiederman M.W.; Pryor T.; Don Morgan C.	1996
740	RELIGIOSITY AND THE SEXUALITY OF WOMEN - SEXUAL-BEHAVIOR AND SEXUAL SATISFACTION REVISITED	DAVIDSON, JK; DARLING, CA; NORTON, L	1995
741	SEXUAL DYSFUNCTION IN MALE-SCHIZOPHRENIC PATIENTS	AIZENBERG, D; ZEMISHLANY, Z; DORFMANETROG, P; WEIZMAN, A	1995
742	SEXUAL FUNCTIONING AND ATTITUDES OF EATING-DISORDERED WOMEN - A FOLLOW-UP-STUDY	MORGAN, CD; WIEDERMAN, MW; PRYOR, TL	1995
743	Incarcerated female sexual offenders: A comparison of sexual histories with eleven female nonsexual offenders	Kaplan, Meg S.;Green, Arthur	1995
744	Sexual dysfunction in male schizophrenic patients.	Aizenberg, D;Zemishlany, Z;Dorfman-Etrog, P;Weizman, A	1995
745	Sexual functioning and attitudes of eating-disordered women: a follow-up study.	Morgan, C D;Wiederman, M W;Pryor, T L	1995
746	Religiosity and the sexuality of women: Sexual behavior and sexual satisfaction revisited	Davidson, J. Kenneth;Darling, Carol Anderson;Norton, Laura	1995

747	Religiosity and the Sexuality of Women: Sexual Behavior and Sexual Satisfaction Revisited	Davidson, J Kenneth, Sr;Darling, Carol Anderson;Norton, Laura	1995
748	Religiosity and the sexuality of women: sexual behaviour and sexual satisfaction revisited	Davidson, J K;Darling, C A;Norton, L	1995
749	Religiosity and the Sexuality of Women: Sexual Behavior and Sexual Satisfaction Revisited	Davidson J.K., Sr.; Darling C.A.; Norton L.	1995
750	Sexual dysfunction in male schizophrenic patients	Aizenberg D.; Zemishlany Z.; Dorfman-Etrog P.; Weizman A.	1995
751	Incarcerated female sexual offenders: A comparison of sexual histories with eleven female nonsexual offenders	Kaplan M.S.; Green A.	1995
752	Sexual functioning and attitudes of eating- disordered women: A follow-up study	Don Morgan C.; Wiederman M.W.; Pryor T.L.	1995
753	MULTIPLE SEX PARTNERS AMONG COLLEGE-WOMEN AND MEN - SEXUAL BEHAVIORS AND SEXUAL SATISFACTION REVISITED	DAVIDSON, JK; DARLING, CA; PENLAND, MR	1994
754	MASTURBATION AND PREMARITAL SEXUAL INTERCOURSE AMONG COLLEGE-WOMEN - MAKING CHOICES FOR SEXUAL FULFILLMENT	DAVIDSON, JK; MOORE, NB	1994
755	Masturbation and premarital sexual intercourse among college women: Making choices for sexual fulfillment	Davidson, J. Kenneth;Moore, Nelwyn B.	1994
756	Masturbation and Premarital Sexual Intercourse among College Women: Making Choices for Sexual Fulfillment	Davidson, J Kenneth;Moore, Nelwyn B	1994
757	Multiple Sex Partners among College Women and Men: Sexual Behaviors and Sexual Satisfaction Revisited	Davidson, J Kenneth, Sr;Darling, Carol Anderson;Penland, Michael R	1994
758	Masturbation and premarital sexual intercourse among college women: making choices for sexual fulfillment.	Davidson, J K;Moore, N B	1994
759	Multiple sex partners among college women and men: Sexual behaviors and sexual satisfaction revisited	Davidson J.K., Sr.	1994
760	African female sexuality and the heterosexual form.	Mcfadden P.	1994

761	Masturbation and premarital sexual intercourse among college women: Making choices for sexual fulfillment	Kenneth Davidson J., Sr.; Moore N.B.	1994
762	MASTURBATORY GUILT AND SEXUAL RESPONSIVENESS AMONG POST-COLLEGE-AGE WOMEN - SEXUAL SATISFACTION REVISITED	DAVIDSON, JK; DARLING, CA	1993
763	GENDER DIFFERENCES IN MASTURBATION AND THE RELATION OF MASTURBATION EXPERIENCE IN PREADOLESCENCE AND OR EARLY ADOLESCENCE TO SEXUAL-BEHAVIOR AND SEXUAL ADJUSTMENT IN YOUNG ADULTHOOD	LEITENBERG, H; DETZER, MJ; SREBNIK, D	1993
764	CHANGES IN SEXUAL-BEHAVIOR FOLLOWING RADIATION-THERAPY FOR CERVICAL-CANCER	KRUMM, S; LAMBERTI, J	1993
765	Sexualität und Bedeutung der Geschlechtszugehörigkeit bei lesbischen und heterosexuellen Paaren. Ergebnisse einer empirischen Studie in den Niederlanden	Schreurs, Karlein	1993
766	Gender differences in sexuality: a meta-analysis.	Oliver, M B;Hyde, J S	1993
767	Gender differences in sexuality: A meta-analysis	Oliver, Mary Beth;Hyde, Janet Shibley	1993
768	Gender differences in sexuality: A meta-analysis	Oliver, Mary Beth;Hyde, Janet Shibley	1993
769	Gender differences in sexuality: a meta-analysis	Oliver, M B;Hyde, J S	1993
770	Gender differences in masturbation and the relation of masturbation experience in preadolescence and/or early adolescence to sexual behavior and sexual adjustment in young adulthood.	Leitenberg, H;Detzer, M J;Srebnik, D	1993
771	Gender differences in masturbation and the relation of masturbation experience in preadolescence and/or early adolescence to sexual behavior and sexual adjustment in young adulthood	Leitenberg, Harold;Detzer, Mark J.;Srebnik, Debra	1993

772	Gender Differences in Masturbation and the Relation of Masturbation Experience in Preadolescence and/or Early Adolescence to Sexual Behavior and Sexual Adjustment in Young Adulthood	Leitenberg, Harold;Detzer, Mark J;Srebnik, Debra	1993
773	Changes in sexual behavior following radiation therapy for cervical cancer.	Krumm, S;Lamberti, J	1993
774	Masturbatory guilt and sexual responsiveness among post-college-age women: sexual satisfaction revisited.	Davidson, J K;Darling, C A	1993
775	Masturbatory guilt and sexual responsiveness among post-college-age women: Sexual satisfaction revisited	Davidson, J. Kenneth;Darling, Carol Anderson	1993
776	Masturbatory Guilt and Sexual Responsiveness among Post-College-Age Women: Sexual Satisfaction Revisited	Davidson, J Kenneth;Darling, Carol Anderson	1993
777	Changes in the Sexual Behavior of Finns 1971-1992	Haavio-Mannila, Elina;Kontula, Osmo	1993
778	Masturbatory guilt and sexual responsiveness among post-college-age women: Sexual satisfaction revisited	Kenneth Davidson J., Sr.; Anderson Darling C.	1993
779	Diabetes mellitus and male sexual function: a controlled study	Schiavi R.C.; Stimmel B.B.; Mandeli J.; Rayfield E.J.	1993
780	Gender differences in masturbation and the relation of masturbation experience in preadolescence and/or early adolescence to sexual behavior and sexual adjustment in young adulthood	Leitenberg H.; Detzer M.J.; Srebnik D.	1993
781	Gender differences in sexuality: A meta-analysis	Oliver M.B.; Hyde J.S.	1993
782	Changes in sexual behavior following radiation therapy for cervical cancer	Krumm S.; Lamberti J.	1993
783	Key variables to understanding female sexual satisfaction: An examination of women in nondistressed marriages	Hurlbert D.F.; Apt C.; Rabehl S.M.	1993
784	THE FEMALE SEXUAL-RESPONSE REVISITED - UNDERSTANDING THE MULTIORGASMIC EXPERIENCE IN WOMEN	DARLING, CA; DAVIDSON, JK; JENNINGS, DA	1991

785	The female sexual response revisited: understanding the multiorgasmic experience in women.	Darling, C A;Davidson, J K;Jennings, D A	1991
786	The female sexual response revisited: Understanding the multiorgasmic experience in women	Darling, Carol A.;Davidson, J. Kenneth;Jennings, Donna A.	1991
787	The Female Sexual Response Revisited: Understanding the Multiorgasmic Experience in Women	Darling, Carol Anderson;Davidson, J Kenneth;Jennings, Donna A	1991
788	Sexual interest, activity, and satisfaction among male nursing home residents	Mulligan, Thomas;Palguta, Robert F.	1991
789	The role of masturbation in marital and sexual satisfaction: A comparative study of female masturbators and nonmasturbators	Hurlbert, David F.;Whittaker, Karen E.	1991
790	The role of masturbation in marital and sexual satisfaction: A comparative study of female masturbators and nonmasturbators	Hurlbert D.F.; Whittaker K.E.	1991
791	The female sexual response revisited: Understanding the multiorgasmic experience in women	Darling C.A.; Davidson J.K.; Jennings D.A.	1991
792	Sexual behavior of inner-city women	House, William C.;Faulk, Alberta;Kubovchik, Margaret	1990
793	Sexual Behavior of Inner-City Women	House, William C;Faulk, Alberta;Kubovchik, Margaret	1990
794	Treating erectile dysfunction with external vacuum devices: impact upon sexual, psychological and marital functioning.	Turner, L A;Althof, S E;Levine, S B;Tobias, T R;Kursh, E D;Bodner, D;Resnick, M I	1990
795	Treating erectile dysfunction with external vacuum devices: Impact upon sexual, psychological and marital functioning	Turner L.A.; Althof S.E.; Levine S.B.; Tobias T.R.; Kursh E.D.; Bodner D.; Resnick M.I.	1990
796	Sexual behavior of inner-city women	House W.C.; Faulk A.; Kubovchik M.	1990
797	Self-perceived differences in the female orgasmic response	Davidson, J. Kenneth;Darling, Carol A.	1989
798	Self-perceived differences in the female orgasmic response.	Davidson, J K;Darling, C A	1989
799	Sexual dysfunction and treatment for early stage cervical cancer.	Schover, L R;Fife, M;Gershenson, D M	1989
800	Sexual dysfunction and treatment for early stage cervical cancer	Schover L.R.; Fife M.; Gershenson D.M.	1989
801	Self-perceived differences in the female orgasmic response.	Davidson J.K.; Darling C.A.	1989

802	Intracavernosal injection in the treatment of impotence: A prospective study of sexual, psychological, and marital functioning	Althof, Stanley E.;Turner, Louisa A.;Levine, Stephen B.;Risen, Candace;Kursh, Elroy D.;Bodner, Donald;Resnick, Martin	1987
803	Intracavernosal injection in the treatment of impotence: a prospective study of sexual, psychological, and marital functioning.	Althof, S E;Turner, L A;Levine, S B;Risen, C;Kursh, E D;Bodner, D;Resnick, M	1987
804	The relationship of sexual satisfaction to coital involvement: The concept of technical virginity revisited	Darling, Carol A.;Davidson, J. Kenneth	1987
805	The Relationship of Sexual Satisfaction to Coital Involvement: The Concept of Technical Virginity Tested	Darling, Carol A;Davidson, J Kenneth, Sr	1987
806	Guilt: A Factor in Sexual Satisfaction	Darling C.A.; Davidson J.K., Sr.	1987
807	Intracavernosal injection in the treatment of impotence: A prospective study of sexual, psychological, and marital functioning	Althof S.E.; Turner L.A.; Levine S.B.; Risen C.; Kursh E.D.; Bodner D.; Resnick M.	1987
808	The relationship of sexual satisfaction to coital involvement: The concept of technical virginity revisited	Darling C.A.; Davidson J.K.	1987
809	Perspectives of sex therapy outcome: A survey of aasect providers	Kilmann P.R.; Boland J.P.; Norton S.P.; Davidson E.; Caid C.	1986
810	Perceived Sexual Satisfaction and Marital Happiness of Bisexual and Heterosexual Swinging Husbands	Dixon, Dwight	1985
811	Perceived sexual satisfaction and marital happiness of bisexual and heterosexual swinging husbands	Dixon, Dwight	1985
812	Perceived Sexual Satisfaction and Marital Happiness of Bisexual and Heterosexual Swinging Husbands	Dixon, Dwight	1985
813	Perceived Sexual Satisfaction and Marital Happiness of Bisexual and Heterosexual Swinging Husbands	Dixon, Dwight	1985
814	Perceived sexual satisfaction and marital happiness of bisexual and heterosexual swinging husbands.	Dixon, D	1985

815	Perceived Sexual Satisfaction and Marital Happiness of Bisexual and Heterosexual Swinging Husbands	Dixon D.	1985
816	Autoeroticism, sexual satisfaction, and sexual adjustment among university females: Past and current patterns	Davidson, J. Kenneth	1984
817	Autoeroticism, Sexual Satisfaction, and Sexual Adjustment among University Females: Past and Current Patterns	Davidson, J Kenneth, Sr	1984
818	Autoeroticism, Sexual Satisfaction, and Sexual Adjustment among University Females: Past and Current Patterns	Davidson, J Kenneth, Sr	1984
819	Autoeroticism, sexual satisfaction, and sexual adjustment among university females: Past and current patterns	Davidson J.K., Sr.	1984
820	Assessment of locus of control: Situational specificity in the sexual context	Catania J.A.; Mcdermott L.J.; Wood J.A.	1984
821	A path analysis model of psychosexuality in young women	Hoon, Peter W.	1983
822	Arousability and sexual satisfaction in lesbian and heterosexual women	Coleman, Emily M.;Hoon, Peter W.;Hoon, Emily F.	1983
823	Sexual fantasy and activity patterns of females with inhibited sexual desire versus normal controls.	Nutter, D E;Condron, M K	1983
824	Arousability and Sexual Satisfaction in Lesbian and Heterosexual Women	Coleman E.M.; Hoon P.W.; Hoon E.F.	1983
825	Sexual fantasy and activity patterns of females with inhibited sexual desire versus normal controls	Nutter D.E.; Condron M.K.	1983
826	A path analysis model of psychosexuality in young women	Hoon P.W.	1983
827	Treatment effectiveness for dysfunctions of sexual desire	Schover, Leslie R.;LoPiccolo, Joseph	1982
828	Treatment effectiveness for dysfunctions of sexual desire.	Schover, L R;LoPiccolo, J	1982
829	Treatment effectiveness for dysfunctions of sexual desire	Schover L.R.; Lopiccolo J.	1982
830	Attitudes of Chinese women towards sexuality and birth control.	Ellis D.; Ho M.S.	1982

831	Human Autoerotic Practices: Studies On Masturbation, edited by Manfred F. De Martino	Goldstein, Bernard	1981
832	A two-dimensional model of female sexual response	Jayne C.	1981
833	Subjective Sexual Experience in College Women	Clifford, Ruth E	1978
834	Massage Parlors and Their Customers	Armstrong, Edward G	1978
835	Styles of Sexual Expression in Women: Clinical Implications of Multivariate Analyses	Hoon, Emily Franck;Hoon, Peter W	1978
836	Sexual enhancement groups for dysfunctional women: An evaluation	Leiblum, Sandra R.;Ersner-Hershfield, Robin	1977
837	Sexual Enhancement Groups for Dysfunctional Women: An Evaluation	Leiblum, Sandra R;Ersner-Hershfield, Robin	1977
838	Sexual enhancement groups for dysfunctional women: an evaluation.	Leiblum, S R;Ersner-Hershfield, R	1977
839	Fatal complication of anal masturbation with a foreign body	Takacs P.; Varszegi Z.; Felegyhazi A.	1977
840	Sexual enhancement groups for dysfunctional women: An evaluation	Leiblum S.R.; Ersner-Hershfield R.	1977
841	Frequency of Sexual Dysfunction in a General Gynecological Clinic: An Epidemiological Approach	Levine, Stephen B;Yost, Murray A, Jr	1976
842	Sexual conseling of the physically disabled.	Anderson T.P.; Cole T.M.	1975
843	Preorgasmic group treatment	Wallace, Douglas H.;Barbach, Lonnie G.	1974
844	Preorgasmic group treatment	Wallace D.H.; Barbach L.G.	1974
845	Psychological aspects of sexual inadequacy in women	Uddenberg N.	1974
846	Aggression and forbiddenness in voyeurism	Yalom, Irvin D.	1960
847	A case of pronounced fetishism	Greenberg, Pearl	1951
848	Some observations on the psychological factors in urination and genitourinary afflictions	Menninger, K. A.	1941
849	A study of sexual tendencies in monkeys and baboons	Hamilton, G. V.	1914

850	A study of sexual tendencies in monkeys and baboons	Hamilton, G. V.	1914
851	A study of sexual tendencies in monkeys and baboons	Hamilton G.V.	1914
